# Emerging capitalism and sociocultural niche construction in early modern Greece

**DOI:** 10.1038/s41467-026-75315-y

**Published:** 2026-07-22

**Authors:** Georgios C. Liakopoulos, Marcus Groß, Piotr Guzowski, Katerina Kouli, Dimitrios Lamprakis, Alessia Masi, Theodoros Vakkas, Elena Xoplaki, Adam Izdebski, Ricardo Fernandes

**Affiliations:** 1 Max Planck Institute of Geoanthropology, Jena, Germany; 2 Department of History and Archaeology, University of Ioannina, Ioannina, Greece; 3 University of Białystok, Białystok, Poland; 4 National and Kapodistrian University of Athens, Athens, Greece; 5 Hellenic Society of Middle Eastern Studies, Halkida, Greece; 6 Ionian University, Corfu, Greece; 7 La Sapienza University of Rome, Rome, Italy; 8 Geospatial Enabling Technologies, Moschato, Attica, Greece; 9 CMCC Foundation - Euro-Mediterranean Center on Climate Change, Lecce, Italy; 10 Institute for Advanced Study, Nicolaus Copernicus University in Toruń, Toruń, Poland; 11 Research Institute for Humanity and Nature, Kyoto, Japan; 12 Department of Bioarchaeology, Faculty of Archaeology, Warsaw, Poland; 13 Masaryk University, Arne Faculty of Arts, Brno-střed, Czech Republic; 14 Princeton University, Climate Change and History Research Initiative, Princeton, NJ USA

**Keywords:** History, Anthropology, Cultural evolution, Socioeconomic scenarios, Environmental impact

## Abstract

Sociocultural niche construction is a key mechanism behind the current planetary crisis. Its causal drivers in past societies remain poorly understood due to a lack of data and modeling complexities. Here, we apply a Bayesian machine learning algorithm to a geospatial quantitative dataset compiled from 15^th^−16^th^ c. CE Ottoman taxation registers, to explore shifts in drivers of land use choices in village communities in southern Greece. Greek and Albanian communities living within the same landscape experienced a transition from unstable political conditions and limited commodification of agriculture to lasting peace, intense market integration and state fiscal pressure, characteristic for early European capitalism. We found that prior to this economic transformation, ethno-cultural identities (Greek or Albanian) played an important role in local ecological niche construction. However, within two human generations (50 years), village communities shifted away from relying on ethnic identities in their ecological choices and their strategies became strongly connected to the investment potential of each village community and the environmental characteristics of their surroundings. Thus, our analysis demonstrates that the pressures of early modern socio-economic systems necessitated highly adaptive behavior from local communities that reduced the ecological significance of traditional identities.

## Abstract (web summary)

Humans constantly transform their natural environments to suit their needs. A case study based on Ottoman taxation data from Greece shows that humans can quickly change the way they shape their environment in response to new economic conditions, abandoning well-established traditions.

## Introduction

Niche construction theory (NCT) describes how organisms modify their environments in ways that enhance their evolutionary success and create lasting ecological inheritances, altering the living conditions for their offspring as well as for those of many other species^1^. Humans are undoubtedly the most powerful among the ecosystem engineers, and the current crisis state of our planetary system results from human niche construction at global scales^2–4^. The deeply sociocultural nature of human activities sets them apart from those of all other species. This phenomenon becomes more evident and complex as human cultural evolution progresses and modern, complex societies emerge. This development not only led to the coordination of human niche engineering activities over greater distances and larger numbers of people, but also to the emergence and spread of new environment-altering technologies at an ever-accelerating pace^5^. Thus, to fully comprehend the mechanisms of this sociocultural niche construction, it is important to extend the prevailing body of relevant research—that so far comes mainly from archaeology and the study of prehistoric or isolated communities^6–9^—to consider empirical data from complex historical societies. Such enrichment of our perspective has the double advantage of studying societies that are more similar to our own in terms of political-economic structures, and of having access to a highly diverse body of evidence that makes it possible to investigate in detail the sociocultural processes and path dependencies that led to present-day modifications of natural environments^10^.

In this paper, we demonstrate how the study of a particularly well-documented complex historical society—that of Ottoman Greece in the 15–16th century CE—reveals a cascading feedback mechanism within a sociocultural system that in the end resulted in significant change (adaptation) of ecological niche construction strategies of peasant farmers. Our case study covers the key period in Europe’s history when the continent’s political economy transitioned from the premodern (medieval) regime to the earliest form of capitalism (15–16th century CE)^11^. These two centuries were characterized by political consolidation within the larger imperial frameworks (Portuguese, Spanish, Austrian, Ottoman, Polish-Lithuanian, to name just a few) and the introduction of innovative administrative solutions that drastically increased fiscal pressures^12,13^. Moreover, Europe underwent a major phase of globalization as well, and economic integration, with the development of intra- and intercontinental trade systems. Simultaneously, there was significant demographic growth, leading to population increases of 30–50% over just a few generations^14^, associated with rapid commodification and monetization of agriculture across the continent^11^.

Our main research question concerns the land use choices of peasant farmers, the actual human ecosystem engineers on the ground, whose actions are also reflected in the palaeoenvironmental records from the region, which we published in other studies^15,16^. The agricultural landscape of the southern Greek countryside during our study period was characterized by a diverse, mixed farming economy, heavily influenced by its challenging environment and land morphology. Even within towns, plots of land were often designated for cultivation, including gardens, vineyards, and even cereal fields; the urban-adjacent areas could not always sustain a town’s full dietary needs^17^. In contrast to the malaria-ridden and swampy lowlands, mountainous areas were often perceived as more desirable locations for human habitation^18^. While cereals, particularly wheat, were a staple cultivated even on slopes and plateaus, this rarely led to monoculture; instead, farmers employed mixed cropping systems alongside viticulture and tree cultivation, which were well-suited to the mountainous terrain. Ottoman historical records reveal diverse sowing proportions, often indicating configurations, such as 50% wheat to 25% each of barley and oats, or, more frequently, 75% wheat to 25% barley^19^. Intensive labor and ingenious land management techniques, such as terracing rugged slopes and constructing irrigation channels which followed contour lines, were essential for maximizing yields. Beyond subsistence crops, proto-industrial cultivations like cotton, flax, and dye-producing plants (such as madder (*Rubia tinctorum L*.) and Aleppo oak (*Quercus infectoria*)) were also significant, often driven by external demand^20^. The cultivated land was primarily divided between cereal fields and vineyards. Fruit trees only showed significant density in specific locations, and their slow growth meant that their soil was not concurrently used for cereal cultivation^21^. Livestock, predominantly sheep, goats, and swine, were vital not only for their direct products but also for their manure, which served as an essential fertilizer for the often nutrient-deficient soil. This was further complemented by an integrated fallowing system and natural deposits from torrents and floods^22^. This holistic approach, integrating various crops and animal husbandry, reflects a pragmatic adaptation to the limited arable land and demanding environmental conditions of Southern Greece.

**Fig. 1:**
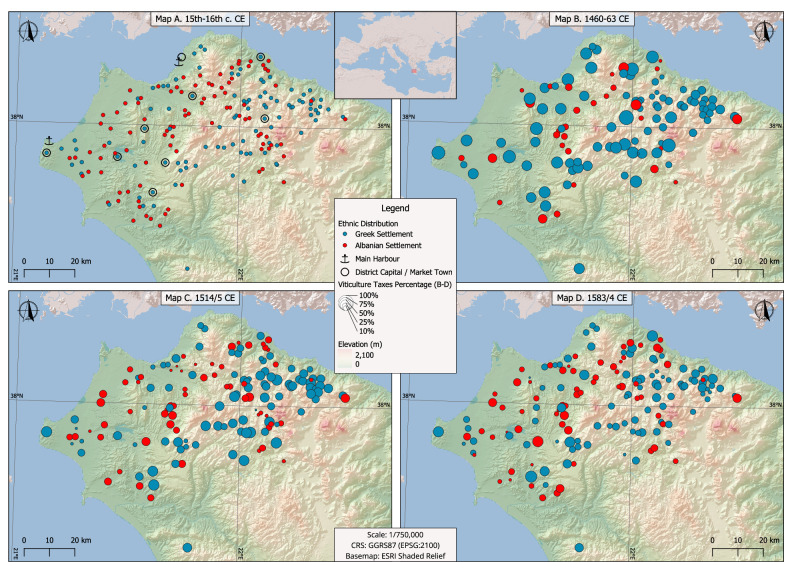
Location and preference for crop trees (vineyards) of the Greek and Albanian settlements recorded in the Ottoman taxation registers available for the northwestern Peloponnese. **A** Spatial distribution of Greek and Albanian villages in the first register, 1460–1463 CE. **B** Viticulture tax percentages in the Albanian and Greek villages in the first register, 1460–1463 CE. **C** Viticulture tax percentages in the Albanian and Greek villages in the second register, 1514/5 CE. **D** Viticulture tax percentages in the Albanian and Greek villages in the third register, 1583/4 CE. *Please note:* some villages did not cultivate viticulture in any of the periods in question and thus appear only on Map (**A**).

In this study, we recover and analyze a unique comprehensive dataset, derived from three consecutive detailed taxation registers produced by the Ottoman administration. They contain information on the population numbers, affluence, and agricultural activities of village communities and are dated to 1460–1463, 1514/5, and 1583/4 CE. Because they reflect conditions characteristic for a given decade, they are here referred to as the 1460s, the 1510s, and the 1580s. For no other part of Europe do we have equally detailed, comprehensive registers that were produced repeatedly over a longer period of time, as were the *taḥrīr defter*s in the 15–16th-century Ottoman Empire^23,24^. Moreover, in our study region of the northwestern Peloponnese in southern Greece, the first register is the only one to contain information on the ethnicity of each community and village (Fig. 1). This is related to the fact that the region previously inhabited by Greek communities experienced migration of Albanian transhumant nomads after the depopulation caused by endemic warfare and recurrent outbreaks of plague in the 14th century CE, and the Venetian and Byzantine authorities that encouraged this migration accorded the Albanians a tax reduction that remained recorded in the first Ottoman register^25^. Crucially, the Greek-speaking populations living in the Peloponnese at the time of the Albanian migration had engaged in mixed agriculture involving in particular grain and crop trees for multiple generations, while the incoming Albanian communities had a strong preference oriented toward wheat cultivation and pastoral farming (see Supplementary Note S1 for detailed information on the Albanian migrations and their ecological aspects).

Historical geography methods were employed to geolocalize most of the villages recorded in the registers. These documents offer information on ethnicity, demographics, affluence (approximated by average agricultural tax per household at village level), and land use choices. While the former phenomena are used here to represent socio-cultural causal drivers of ecosystem niche construction choices, the latter (land use choices) are employed to approximate the niche construction strategy. Specifically, we are interested in the degree to which village communities engaged in tree-crop versus grain- and livestock-focused agriculture. This represents the broad spectrum of possible ecological niche construction strategies available in the Mediterranean environments^26^. In addition, we compiled environmental variables that may represent different causal drivers of human niche construction in our study region (see Methods).

Here, we hypothesize that the transition to the early capitalist political economy resulted in a rapid and profound change in the dominant causal drivers of human niche construction in the study region, reflecting the capacity of humans to adapt to new sociocultural pressures by reshaping their actual ecological niche^27,28^. This hypothesis departs from the findings of the previous studies on human niche construction, which tend to emphasize the stabilizing influence of social learning and cultural identities (cultural inheritance) on ecological niche construction by human communities^6–8^. To the contrary, we expect that when villagers found themselves under increasing market and fiscal pressures, they were rapidly reorienting themselves, changing both their agricultural choices and their framework of reference in ecological-economic considerations. Establishing causal structures in complex adaptive systems is a difficult task, particularly when dealing with limited historical-observational data. We employed a Bayesian machine learning method (BMSC: Bayesian Model Selection under Constraints) to explore temporal changes in variable associations and discuss how the modeling results align with our causal hypothesis and the expected impacts of modeling variables.

## Results

The BMSC algorithm was used to compare how different regression models explained the differing involvement of Greek and Albanian communities in crop-tree agriculture (see Supplementary Note S1). The degree of village involvement in crop-tree agriculture, understood as a niche construction strategy, was represented by a numeric independent variable representing the share (in %) of vineyard taxes in the total cultivation taxes of each village (please note that grain and vine were subject to comparable tax burden in the Ottoman system, see Supplementary Note S2). As vine was the key tree crop in the specific bioclimatic conditions of the northwestern Peloponnese^29–32^, the relative importance of vine cultivation in each village—as reflected by taxation data—reflects the extent to which a given village engaged in tree-crop intensive agriculture as opposed to more extensive grain-and-herding-focused agriculture. The latter, in particular, was characteristic of the Albanian transhumant nomads in the initial phase of their settlement in the Peloponnese (see Supplementary Note S1). Moreover, as grain was taxed in kind and vine products primarily in cash, the extent of a village's involvement in vine cultivation also reflects the degree of commercialization of its economy. As shown in Fig. 1B–D, there occurred significant changes in the distribution of vine cultivation in our study region between the 1460s and the 1580s. While in the 1460s (Fig. 1B) it was predominantly the Greek villages that engaged in agriculture, already in the 1510s (Fig. 1C) this pattern changes, with some Albanian villages appearing on the map for the first time: they only started engaging in vine cultivation in the half century between the 1460s and the 1510s. This picture becomes even more complicated in the 1580s (Fig. 1D). It is this changing pattern of the distribution of vine cultivation—i.e., of niche construction involving a significant proportion of crop-tree agriculture—in the northwestern Peloponnese that we attempt to explain in this study.

**Fig. 2:**
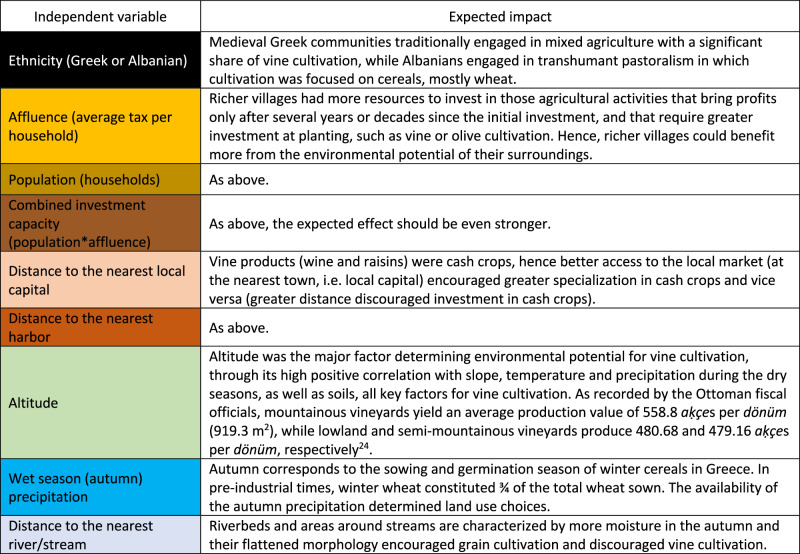
Independent variables considered by the Bayesian machine learning algorithm. Expected impact on the dependent variable.

**Fig. 3:**
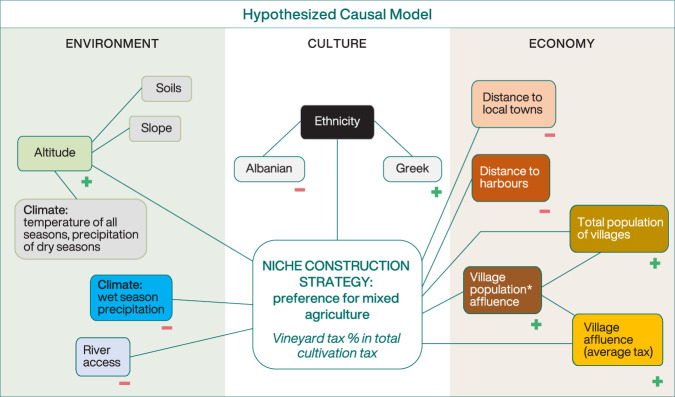
Hypothesized causal model of human niche construction in the early modern northwestern Peloponnese with the expected direction of impact. Environmental variables strongly correlated with the altitude are shown in gray and not included in model calculations. For the details, please look into Fig. 2. Colors follow the same pattern as Fig. 6.

To constrain the selection of dependent variables to be included in the BMSC, we first assessed the correlation among a wider set of variables. The results are given in Table S2. In the particular geomorphological context of the northwestern Peloponnese, environmental variables—soil types, slope, mean temperature of all four seasons, and precipitation totals in winter, spring, and summer—are strongly correlated with the altitude. These dependencies imply that the added explanatory value of these variables is limited and may lead to overfitting and misleading interpretation of the results. Consequently, we did not include them in the model, and we do not discuss their expected causal impact in Fig. 2 (but we show them in Fig. 3). Rather, we only included altitude as an explanatory environmental variable. In the BMSC model comparison, we considered up to the second-order exponents for each variable and the interactions among the different orders for population and affluence, representing village investment capacity. The cultivation of crop trees required larger initial investment, altering the local ecological niche—land formation into terraces or plantations, planting of vines, building supports—than cultivating cereals or herding livestock, and the first returns on the investment could only come after a few years, increasing over time^30^.

**Fig. 4:**
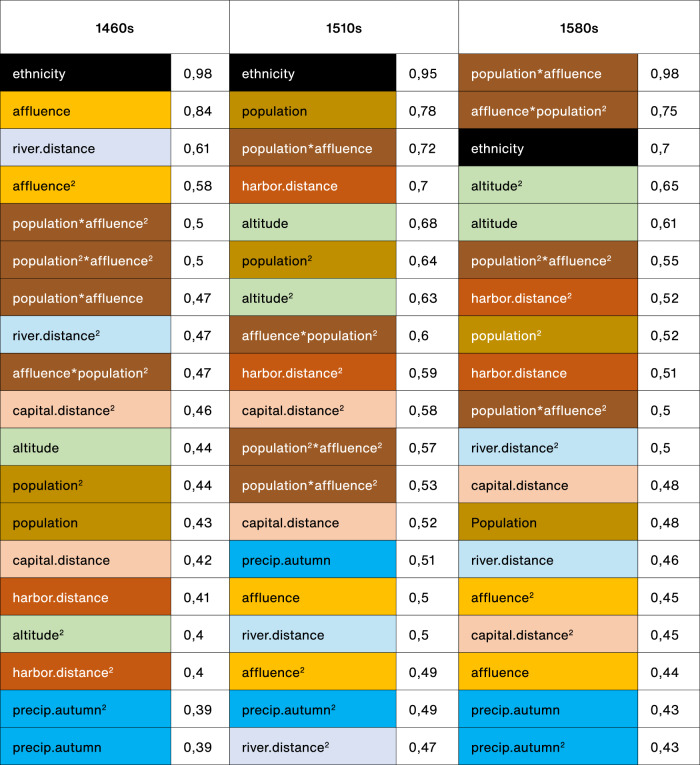
Global variable importance calculated by the machine learning algorithm BMSC. Based on standardized coefficient estimates of all the models calculated by the algorithm. Colors based on Figs. 2, 3.

BMSC was run for data from each decade (1460s, 1510s, 1580s). For each decade, regression models were compared and ranked according to Bayesian R^2^ and the Bayesian information criterion (BIC), and the different variables were ranked according to their global variable importance (see Methods for more details). The results for the global variable importance for each decade are given in Fig. 4. They reveal that village ethnicity (Greek vs Albanian) was the most important variable in the 1460s and the 1510s, but was overcome by the village investment capacity in the 1580s. The process of village investment capacity becoming the most important of the economic variables was already underway in the 1510s; at that time, the distance to harbors and village population also played a prominent role. As for the environmental variables, there is a difference between the 1460s, when the distance to the nearest river/stream (representing environmental conditions relevant for cereal cultivation) is one of the three higher-ranked variables, while in the 1510s, and even more in the 1580s, altitude (representing environmental conditions relevant for vine cultivation) becomes the key environmental variable. Finally, there is more similarity in the ranking pattern of the global variable importance between the 1510s and the 1580s than between any of these decades and the 1460s. This suggests that the importance of change between the first and the second decade was larger than between the second and the third.

**Fig. 5:**
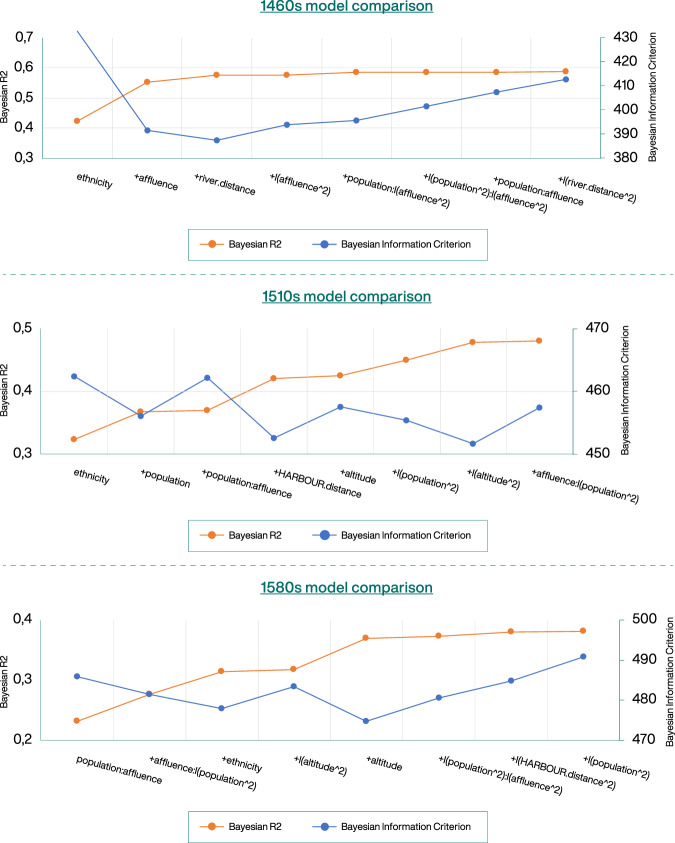
Distribution pattern of Bayesian R2 and Bayesian Information Criterion of 8 optimal models selected by the machine learning algorithm BMSC for each of the three registers. TT10-1/14662 from 1460-63 CE, TT80 from 1514/5 CE, and TT607 from 1583/4 CE.

The Bayesian R^2^ and BIC for the eight best models are shown in Fig. 5 (see Table S1 for further details). Bayesian R^2^ values for all three decades are satisfactory, meaning that our independent variables explain a significant amount of the dependent variable’s variance. However, they decline with time, suggesting that our independent variables were losing their explanatory power over time (the highest Bayesian R^2^ is that of the 1460s, i.e., ca 0.58, while the 1510s decline to ca 0.47 and the 1580s to ca 0.37). Regression models in Fig. 5 are ranked according to Bayesian R^2^ and BIC values, and hence it is visible that for each decade the models placed beyond the model with the lowest BIC value achieve only incremental increases in Bayesian R^2^: they increase complexity with little gain in explanatory power, leading to model overfitting. It is also worth noticing that, again, there are similarities, this time in the R^2^ and BIC distribution patterns, for the 1510s and 1580s, while the results for the 1460s stand out, as was also the case for the global variable importance.

The best models according to BIC values are shown in Table 1. For each of the three decades, Table 1 presents the model intercept, the regression coefficient estimates for independent variables (with median, standard deviation, and credibility intervals) as well as the standardized coefficients. These coefficients reflect the importance of independent variables in the regression model. Here, in Table 1, the decline of the importance of ethnicity over time is even more visible than in the global variable importance rankings (as discussed above, Fig. 4). Whereas in the 1460s the Greek villages are predicted to have the vine tax proportions higher than the Albanian villages by ca. 21 percentage points by mere virtue of their ethnicity, this “Greek ethnicity premium” declines to ca seven percentage points in the 1510s and just ca. three points in the 1580s. Moreover, the village affluence and the distance to the nearest river/stream, which were the key variables in the 1460s, completely disappear from the best models in the 1510s and the 1580s. Finally, whereas altitude remained the main variable in both the 1510s and the 1580s, in the 1580s the village investment capacity took over the position occupied by the village population in the 1510s. Thus, again, a major change occurs between the first and the second decade, while the patterns visible in the second decade are further reinforced in the third decade.

**Table 1:** Model summary of the best model for each register (TT10-1/14662 from 1460-63 CE, TT80 from 1514/5 CE and TT607 from 1583/4 CE). Selected on the basis of the Bayesian Information Criterion (Fig. 5)

Parameter	Importance (standard. coefficient)	Estimate / Value (Sigma, Log)	Median	SD	Credibility Interval
1460s CE					
(Intercept)		-14.300	-14.20000	2.860000	[-19.9, -8.66]
Ethnicity (Greek)	0,51	21.2000	21.20000	2.150000	[17, 25.4]
Affluence	0,38	0.32600	0.32500	0.044900	[0.237, 0.414]
river.distance	0,16	0.00299	0.00298	0.000938	[0.00114, 0.00483]
Sigma		13.5000			[12.2, 15]
Log-likelihood		-183.379			

1510 s					
(Intercept)		-4.45000	-4.450000	2.30000	[-8.94, 0.0887]
altitude	0,72	0.027400	0.027400	0.00818	[0.0114, 0.0435]
population	0,67	0.165000	0.165000	0.04890	[0.0699, 0.261]
altitude^2^	0,67	-0.000023	-0.000023	0.00000714	[-0.0000371, -0.0000091]
population^2^	0,48	-0.000487	-0.000487	0.000156	[-0.000793, -0.000181]
Ethnicity (Greek)	0,28	7.350000	7.350000	1.87000	[3.67, 11]
harbor.distance	0,19	0.000172	0.000172	0.0000547	[0.0000648, 0.000279]
population*affluence	0,06	0.000122	0.000123	0.000234	[-0.000341, 0.000582]
Sigma		9.77000			[8.81, 10.9]
Log-likelihood		-204.931			

1580 s					
(Intercept)		2.33000000	2.32000000	1.74000000	[-1.13, 5.75]
altitude^2^	0,89	-0.0000248	-0.0000248	0.00000628	[-0.0000371, -0.0000125]
altitude	0,89	0.02730000	0.02730000	0.00709000	[0.0134, 0.0413]
population*affluence	0,85	0.00128000	0.00128000	0.00022200	[0.000846, 0.00172]
affluence*population^2^	0,45	-0.00000316	-0.00000316	0.00000100	[-0.00000513, -0.00000118]
Ethnicity (Greek)	0,14	3.00000000	3.0000000	1.45000000	[0.143, 5.84]
Sigma		8.700000			[7.85, 9.67]
Log-likelihood		-221.778			

In the 1510s and the 1580s, altitude and village population appeared in the models with both their first and second exponent. In the case of village population, it also interacts with affluence, the interaction reflecting the relative investment capacity of a village in the 1580s. As regards the direction of the impact of the independent variables, in almost all the cases it is as predicted in Figs. 2, 3, except for the distance to the nearest harbor in the 1510s (it was predicted as negative, but turned out to be positive).

Assessments of the convergence of the models are reported in Tables S3. The best-ranked model according to the BIC for each time period (1460s, 1510s, 1580s; Table 1) passed the convergence tests employed in BMSC modeling.

## Discussion

The best models selected by the BMSC for the three decades (1460s, 1510s, 1580s) show a good degree of explanatory power, although this declines with time. Moreover, these models differ, and as we know that the recording procedures for the cadasters remained relatively stable, this change should be related to the change in how human niche construction was organized.

Overall, modeling results reveal variable associations that align with our hypothesis, including the direction of impact of the different independent variables, with one exception, that of the distance to the nearest harbor. Based on research showing a positive role of access to transport networks as an incentive for market-oriented specialization of agriculture^33^, we expected that closer proximity to harbors encouraged cultivation of the vine, being a better export-oriented cash crop. Thus, we anticipated a negative relationship between the distance and the dependent variable of vineyard preference. It turned out, however, that when it did matter, this variable relationship was positive: the farther away from the harbor, the more likely the villagers were to engage in vine cultivation, even if the effect was relatively minor compared to the other independent variables included in the model for the 1510s. This can be explained by the economic density of the products of vine cultivation: per unit of weight or volume, which dictated transport costs and technical difficulty in the mountainous terrain of the northwestern Peloponnese, wine was valued more than grain (see Supplementary Note S3 for references and detailed discussion). Thus, obtaining monetary surplus from agriculture, necessary under conditions of strong market and fiscal pressures (which for the Albanians increased over time, as their poll tax reduction of 20% was abolished in the 1510s), was more easily achieved (for the villages located away from the major trade hubs) when it came to transport and trade logistics by selling wine products rather than grain.

**Fig. 6:**
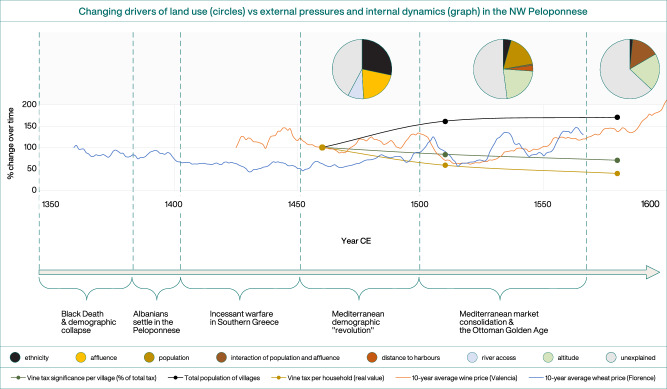
Relative importance of associated drivers for each (circles) register against relative changes in globalized market pressure and internal dynamics of the studied region, based on the best model indicated by Bayesian Information Criterion (Fig. 5). The gray area within the pie charts is the unexplained R^2^. The size of the pies is proportional to the share of the standardized coefficients for each variable in the sum of all standardized coefficients included in the best model. Sources of data: vine tax significance, total population, and vine tax per household come from the Ottoman taxation registers discussed in this study; Mediterranean price indices were calculated on the basis of available data^61^.

Figure 6 shows the explanatory power of the best model for each decade (1460s, 1510s and 1580s) and the ranking of variables within each model against the backdrop of regional political/economic history and the general economic trends in the Mediterranean. These trends are expressed in wine and wheat prices from the major trade centers of Valencia in Spain and Florence in Italy, the longest available series of that kind. This wider multi-continental context of the Mediterranean is particularly relevant, as our study region of the northwestern Peloponnese was increasingly integrated in the broader markets that drove the demand for cash crops, such as wine or grain^34–36^. Moreover, as price records become available from the late 15th century onward for the Ottoman Empire, to which most of the Peloponnese belonged at the time, they show very close positive correlation with the Italian prices, in particular for grain^37^.

Both the wine and the grain price series from the Mediterranean reveal that the demand for these products was growing fast in the second half of the 15th century, and well into the 1500s. This was exactly the period when the likely causal drivers shifted from cultural and grain-focused (ethnicity and river/stream distance in the 1460s) to those related to the cultivation of the more profitable cash crop, vine (altitude, affluence, harbor access, and investment capacity in the 1510s). Interestingly, this change occurred despite the fact that the general prevalence of vine cultivation in the agricultural portfolio of the villages in our region slightly declined, which can be explained by the need to intensify cereal cultivation to feed the growing population, as well as the further diversification and specialization of agriculture. As the prices continued to rise, punctuated by periods of crises, and the Mediterranean trade integration strengthened even further, the independent variables related to the environmental and economic potential of villages became even more important in the 1580s. In other words, what we see is not necessarily the intensification of vine cultivation, but a change in how villagers make decisions that shape their ecological niche: instead of relying on social learning (Albanian or Greek ethnic identities determining the available ecological knowledge, as inherited from previous generations), they responded to what economic and environmental potential their local environment represented in the newly emergent capitalist conditions.

We can reconstruct this process in even more detail. As shown in Fig. 6, our first register is available from ca 70 years after the initial settlement of the Albanians in the Peloponnese (see Supplementary Note S1). This suggests that during this long initial period the Albanians persisted in their original ecological niche construction strategy, focused on cereals and transhumant pastoralism, which in the earliest register (1460s) is evident in the highest importance of ethnicity as the variable associated to peasants’ ecological choices and may be related to the different demographic structure of Albanian and Greek households, extended versus more nuclear, respectively (see also Supplementary Note S1). This period of persistent human niche construction strategy falls into times of incessant warfare, limited market integration, and overall political-economic uncertainty in the Peloponnese. The biggest change, the adaptive shift in niche construction from cultural and ecological inheritance to responsiveness to environmental and economic potential, occurs during a peaceful period of unprecedented political-economic consolidation, under the nascent capitalism^11^. This time period, for its fast pace of all-encompassing change as well as the rapid and ubiquitous demographic growth, is known as the Mediterranean demographic “revolution”^14^. Also during that time, we see capital accumulation among peasant families in the villages of our study region, a process that is characteristic of a capitalist economy: there is a change in the regional distribution of average household agricultural tax at village level (reflecting village affluence, as used in modeling) toward higher values and greater variation (Supplementary Information Fig. S2).

During the Ottoman “Golden Age” of the 16th century, when the empire of the Istanbul Sultans ruled over half of the Mediterranean and was one of the dominant military powers in western Eurasia, the profound change in the drivers of ecological niche construction that occurred in the later 15th century consolidated even further. What is striking, this major shift in the prevailing drivers behind human niche construction in our study region did not lead to the disappearance of the ethnic identities that were still so important for human ecological choices in the middle of the 15th century The Albanian ethnic identity in the Peloponnese is well attested in the subsequent centuries, up until the 20th century^38–40^ In this context, it should be noted that vine cultivation and wine production require specialized knowledge, which was not part of the traditional Albanian lifestyle. This means the Albanians needed to learn that from the neighboring Greek communities, which may have occurred through direct contact or with the help of the *timariots* (the noblemen who received taxation payments from the villagers as part of their remuneration for the military service to the sultan) and their managers.

In more general terms, the historical communities whose ecological niche construction strategies we study in this paper demonstrated highly agile adaptive behavior when faced with external economic and fiscal pressures. This discovery nuances earlier research on the role of within-group social learning and cultural-ecological path dependency^6–8^. As the needs and opportunities arose, creating cascading feedback in the sociocultural system, our villagers adapted rapidly and changed their niche construction strategies by making the most of the environmental and demographic-economic potential that was available to them, rather than sticking to traditional practices. Under the conditions of the emerging European capitalism, the causal drivers behind the niche construction strategies in southern Greece shifted almost entirely from culture- and subsistence-driven to profit-oriented.

At the same time, it is highly unlikely that the observed changes in variable associations are related to climate variability, even during the Little Ice Age. While paleoclimate proxies for southern Greece in the 15–16th century are lacking, two important proxies (sea surface temperature^41^ and high elevation June-July precipitation derived from tree rings^42^) are available for northern Greece. While the sea surface temperature indicates overall stable climatic conditions in the northern Aegean region over the 15–16th century after a shift towards more humid conditions during the cool season of the year from mid-15th century, the tree-ring reconstruction suggests some increase in early-summer dryness in the final decades of the 15th century. The Old-World Drought Atlas reconstruction for southern Greece, Fig. S1, which reflects winter-spring drought conditions combining temperature and precipitation conditions^43^, shows only a minor shift, which was unlikely to have a major impact on the cultivation and investment decisions of the Greek and Albanian villages. Thus, our case study indicates that intertwined sociocultural processes on multiple levels of social hierarchy and organization, independent from natural environmental pressures, have the potential to strongly influence human ecological niche construction activities “on the ground”, in particular landscapes and ecosystems.

Some limitations, nevertheless, should also be considered when interpreting our results. The Bayesian modeling approach we employ identifies associations between variables rather than establishing causal mechanisms, and the declining Bayesian R² values across the three periods suggest that unmeasured factors, such as local land-tenure arrangements, the role of intermediary Ottoman officials, or micro-level social dynamics, played an increasingly important role over time. Specifying a fully causal model is, in any case, inherently challenging when dealing with a complex adaptive system, in which cultural identities, economic incentives, environmental constraints, and institutional structures interact through non-linear feedback loops that evolve; imposing a rigid causal structure would risk oversimplification and a false sense of mechanistic certainty. Within these constraints, however, our approach offers distinct advantages. The association-based BMSC framework allows the data to reveal shifting patterns of variable importance without forcing them into a predetermined causal hierarchy, remaining agnostic about directionality in a setting where reciprocal causation is almost certainly at play. Moreover, the signal in the ethnic pattern of vine cultivation is robust, despite the potential limitations of the source material, such as inconsistencies in fiscal recording practices, incomplete source survival, uncertainties in geolocating historical settlements, and the simplifying assumptions inherent in using tax categories as proxies for on-the-ground agricultural realities. In the 1460 s register, vineyard taxes are overwhelmingly concentrated in Greek villages, with Albanian communities showing virtually no engagement in viticulture, yet a mere two generations later Albanian villages appear as active vine cultivators, and by the 1580s the previously sharp ethnic divide has largely dissolved. That the same fiscal system, administered by the same imperial bureaucracy, captured this convergence across three sequential cadasters spanning over 120 years strongly suggests genuine behavioral change rather than documentary noise. Our modeling thus captures the evolving structure of this socio-ecological system in a transparent and epistemologically honest manner, demonstrating robust, temporally evolving associations that are consistent with our proposed framework without overclaiming what observational historical data can deliver.

To conclude, our research reveals that while the human ecological niche construction strategies remain stable and resist adaptation under conditions of insecurity, a more predictable political-economic framework combined with strong fiscal and market pressures encourages innovation, experimentation, and flexible adaptation. The processes visible today across the globe, where combined pressures of the markets and the state force rural communities to abandon traditional lifeways and rapidly transform ecosystems to maximize profits, clearly operated half a millennium ago in Europe, resulting in a regime shift: from medieval to modern. Such rapid processes can lead to both positive and negative outcomes for the overall sustainability and biodiversity preservation^44^. Moreover, our research demonstrates that paradoxically there is a greater chance of achieving lasting change under predictable and stable conditions, such as those that characterized the Ottoman Empire from the late 15th century, than under the conditions of insecurity that discouraged innovation and investment, and which characterized our study region in the preceding century. These potential lessons demonstrate the direct relevance of interdisciplinary historical research, with its longer time-scales and broader range of comparative cases, for modern policy questions^45,46^.

## Methods

### Model variables

#### Ethnicity

The first extant Ottoman detailed taxation register of the Peloponnese has survived in a fragmentary state and was divided in the early 1930s into two parts: (a) the TT10, housed in the Prime Ministry Ottoman Archives in Istanbul and (b) the 1/14662, housed in the St. St. Cyril and Methodius National Library of Bulgaria in Sofia^25^. This manuscript, dated 1460–63, divides the recorded settlements into Greek and Albanian based on different rates of *per capita* taxation, which favored the Albanians. We are justified in supposing that this differentiation dated from an earlier period and reflected the services rendered to the state at the time of the Albanians’ initial settlement^47^. The Albanians immigrated to the Peloponnese in the second half of the 14th century, with the two main waves of influx dated in the late 1390s and the late 1410s^48^. The posterior registers, TT80 (1514/5) and TT607 (1583/4), do not ethnically distinguish the settlements, as the same amount of tax was levied on both Greek and Albanian subjects, but given the persistence of the Albanian culture in the Peloponnese into the 19–20th century, we assume the stability of these village ethnic identities also in the 16th century^48^, which is corroborated by an anthroponymic study on the recorded villagers. Also, in the TT10, there was only one ethnically mixed village, Martina der Noḳastro, predominantly Greek already at that point (10 to 5) and almost exclusively in TT80 (32 to 1) and TT607 (27 to 2). In our calculations, we take only the data on the Greek households from this village.

#### Vineyard tax percentage

Percentage of taxes in *aḳçe*s (Ottoman silver coin) imposed on *reʿāyā* villagers’ and *timariots*’ personal demesne vineyards (*bāġāt-ı ḫāṣṣa*) among cultivation taxes in registers TT10-1/14662 (1460–1463), TT80 (1514/5) and TT607 (1583/4).

#### Households (village population)

Christian and Muslim adult male-headed hearths (*ḫāne*) and small hereditary possessions (*baştina*) in registers TT10-1/14662 (1460-63), TT80 (1514/5) and TT607 (1583/4).

#### Average tax (village affluence)

The fraction of the total amount of taxes levied on growing crops and raising livestock of the *reʿāyā* villagers in a given village in *aḳçe*s *per annum*, divided by the number of households in the village, in registers TT10-1/14662 (1460-63), TT80 (1514/5) and TT607 (1583/4). It excludes *per capita*, market and craft industry taxes, dues, and fines.

#### Altitude

Altitude values have been obtained from a raster digital elevation model (former Greek National Mapping and Cadastre Organization, Association of Rural and Surveying Engineers). The physiographic distribution of the Peloponnesian land is as such: mountainous ( > 700 m), semi-mountainous (100–700 m) and lowland (0–100 m)^49^.

#### Soils

The soils layer has been drawn after the location map of selected soil profiles in Greece^49^.

#### Slope

The slope factor was determined by calculating zonal statistics, using a 5 m resolution slope raster dataset against a 5 km buffer zone around each settlement point.

#### Temperature, precipitation

To approximate the difference in local microclimate between the villages, we considered the mean climate conditions derived from the 20th century climate data. For each of the four seasons, we acquired mean precipitation totals and temperature values from the Climatic Atlas of Greece (http://climatlas.hnms.gr; time period of data acquisition: 1977–2000). The autumn season of September, October, and November corresponds to the sowing season of winter cereals in Greece, i.e., October for mountainous regions and November for the plains^50,51^. Winter wheat constituted ¾ of the total wheat sown in mid-19th-century Greece^52–56^.

#### Distance to the local capital

Euclidean distance between each village and the nearest local capital or market town, marked as district capital (*nefs*) in the TT10-1/14662 tax register, that is: Ayo İlya (Agios Ilias), Balya Badra (Patras), Ġardicḳo (Gatsiko), Girbene (Spartia), Ḫulumiç (Hlemoutsi), Ḳalandriça (Halandritsa), Ḳalavrita (Kalavryta), Ṣandamiri (Santomeri), Voştiça (Aigio), and Vumero (Goumero). As a result of a new, more centralized administrative division that must have taken place at the turn of the 16th century and is portrayed in the TT80 and TT607, the district of Balya Badra incorporated the districts of Girbene, Ḳalandriça, and Ṣandamiri; the district of Ḳalavrita incorporated the one of Voştiça; and the district of Ḫulumiç incorporated the ones of Ayo İlya, Ġardicḳo, and Vumero. The district of Salmenik (Ano Salmeniko) is not included in the extant folios of the TT10-1/14662; it is only mentioned with reference to its warden, timariot Ayas (TT10, 184). In the TT80 and TT607, it merged with the district of Balya Badra. Hence, it is not included in the present study.

#### Distance to the nearest harbor

Euclidean distance between each village and the nearest main harbor, which for the late medieval and early modern northwestern Peloponnese was Glarentza and Patras^55^.

#### Data on altitude difference for harbors and local towns

In an additional attempt to assess the access factor of the villagers to the local capital/market, the maximum differentiation in altitude between each settlement and the nearest capital was calculated by profiling the straight segment connecting using a 30 m sampling distance. However, it was not included in modeling as it proved to have high correlation with the altitude variable. The same applies for the distance to the nearest harbor.

#### Distance to the nearest river/stream

Euclidean distance between each village and the nearest running water. Shapefile hydrography layer obtained from the Hellenic Mapping and Cadastral Organization 1:50,000 (http://geodata.gov.gr/).

All the geographical data and the ethnicity were the same for all three model runs (1460s, 1510s, 1580s). Data correlation table for independent variables from Fig. 2 and Fig. 3 are available in the supplementary information (Table S2). The dataset itself is available on the Pandora data platform via the “Environmental History” data community and the “Tax records for Ottoman Peloponnese” repository: 10.48493/rz8t-wv22.

### Bayesian model selection under constraints (BMSC) machine learning algorithm

Bayesian Model Selection under Constraints (BMSC) is an algorithm designed to pinpoint the most effective set of variables for explaining and predicting a particular dependent variable. BMSC employs a Bayesian two-step process to sift through a vast array of potential models. It considers polynomial and exponential terms—up to a power or depth of three—as possible variables (in our modeling, we used the power and depth of two, however).

A comparable method is the Deletion/Substitution/Addition (DSA) algorithm^56,57^, which utilizes a stepwise process to determine the optimal model. However, BMSC differentiates itself by incorporating a Bayesian perspective in variable selection. This approach has been shown to perform better than frequentist approaches in accurate variable ranking^58^. Central to the Bayesian approach is the horseshoe prior method^59^, which adeptly merges model selection, shrinkage (or regularization), and variable importance ranking. This method produces a shrinkage weight, denoted as ‘K’, ranging from 0 to 1. Values closer to 1 suggest a variable’s significance, indicating it should be included in the model with minimal shrinkage by the horseshoe prior, and vice versa.

In BMSC’s initial step, ‘K’ is calculated for each candidate variable, which includes the selected variables along with their interaction depth and exponential terms. This ‘K’ serves as a universal measure of variable importance. To avoid introducing regressor shrinkage, a second step is undertaken where standard Bayesian linear models are fitted. This is done sequentially, starting with the feature having the highest ‘K’ value, followed by the next highest, and so on.

Subsequently, these models are evaluated using various fit criteria, such as leave-one-out cross-validation, Watanabe-Akaike Information Criterion (WAIC)^60^, Bayesian Information Criterion (BIC), (Bayesian) R-squared, or log-likelihood. This comparative analysis aids together with (Bayesian) model and variable diagnostics and an alternative for measuring the variable importance (“standardized coefficients”) to the ‘K’ value in selecting the most suitable model.

The full R-code for BMSC, including access to a corresponding online app, is available at the Pandora & IsoMemo software repository (10.48493/rz8t-wv22). Modeling was conducted using a local version of BMSC by setting inputs via a graphical interface (BMSCS: https://github.com/Pandora-IsoMemo/BMSCS). Each model file is available at the “Tax records for Ottoman Peloponnese” repository: 10.48493/rz8t-wv22. A Readme file, available at the same repository, describes the use of BMSCS.

Before modeling, we checked for potential spatial autocorrelation within each of our three separate temporal datasets; it proved to be either non-significant (1460s, 1580s) or weak (1510s) (Supplementary Note S4 and S5).

To all three register datasets, we applied the following settings: interaction depth 2, maximal exponent 2, excluding all interactions other than the four interactions of village population and affluence. To ensure the robustness of the results, we set the number of MCMC chains to eight (maximum), burn-in iterations to 2000, and MCMC iterations to 10,000. Model convergence was overall satisfactory following assessments using Gelman Scale Reduction Factor, Raftery and Lewis, Geweke z-Score, and Heidelberger-Welch tests. Model outputs, including diagnostics, are available (in xlsx format) at 10.48493/rz8t-wv22. Since our dependent variable is constrained between 0 and 100%, we checked if model predictions were within bounds. For the 1460s dataset, only 6% of predictions were slightly out of bounds (below 0%), while for the 1510s and 1580s datasets there were no predictions out of bounds (Supplementary Information Figs. S3–S5).

### Reporting summary

Further information on research design is available in the Nature Portfolio Reporting Summary linked to this article.

## Supplementary information

Supplementary Information

Reporting Summary

Transparent Peer Review file

## Data Availability

The underlying dataset is available on the Pandora data platform via the “Environmental History” data community and the “Tax records for Ottoman Peloponnese” repository: 10.48493/rz8t-wv22.

## Code availability

The full R-code for BMSC, including access to a corresponding online app, is available at the Pandora & IsoMemo GitHub software repository with link for this and implemented model instances within the context of this work available at: 10.48493/rz8t-wv22.
