# Supplementary material for: Emerging capitalism and sociocultural niche construction in early modern Greece
Source: Nat Commun. 2026 Jul 22;17:7119. doi: 10.1038/s41467-026-75315-y (PMC13396767; doi:10.1038/s41467-026-75315-y)
Supplement: Supplementary file 1 — Supplementary Information [file 41467_2026_75315_MOESM1_ESM.docx]

**Emerging capitalism and sociocultural niche construction in early modern Greece**

**Supplementary Information**

Georgios C. Liakopoulos^1,2^*, Marcus Groß^1^, Piotr Guzowski^3^, Katerina Kouli^4^, Dimitrios Lamprakis^5^, Alessia Masi^1, 6^, Theodoros Vakkas^7^, Elena Xoplaki^8^, Adam Izdebski^1, 9, 10^*, Ricardo Fernandes^1, 11,12,13^*

^1^ Max Planck Institute of Geoanthropology, Jena, Germany

^2^ Department of History and Archaeology, University of Ioannina, Greece

^3^ University of Białystok, Poland

^4^ National and Kapodistrian University of Athens, Greece

^5^ Hellenic Society of Middle Eastern Studies, Halkida, Greece

^6^ La Sapienza University of Rome, Italy

^7^ Geospatial Enabling Technologies, Moschato, Attica, Greece

^8^ CMCC Foundation - Euro-Mediterranean Center on Climate Change, Italy

^9^ Institute for Advanced Studies, Nicolaus Copernicus University in Toruń, Poland

^10^ Research Institute for Humanity and Nature, Kyoto, Japan

^11^ Department of Bioarchaeology, Faculty of Archaeology, Warsaw, Poland

^12^ Masaryk University, Arne Faculty of Arts, Brno-střed, Czech Republic

^13^ Princeton University, Climate Change and History Research Initiative, Princeton, USA

Corresponding authors: Georgios C. Liakopoulos (liakopoulos@gea.mpg.de), Adam Izdebski (izdebski@umk.pl).

* These authors contributed equally.

**Supplementary information contents:**

Supplementary Notes S1-S5

Figures S1-S5

Tables S1 to S3

SI References

**Supplementary Note S1**

**The Albanian migration into the Peloponnese**

The Ottoman Empire was a multi-ethnic and multi-continental monarchy that dominated southeastern Europe, southwestern Asia and northern Africa from the 15th until the early 20th c. CE. It was characterized by a developed market economy and a highly complex and effective taxation system based on poll and agricultural production taxes. In the Peloponnese of the 15th c., while the agricultural taxes were equal for all ethnic groups, the poll tax (*ispence*) was slightly lower (20%) for the Albanian population, and hence the ethnic identity of each village recorded in the taxation register of 1460-63 CE was noted down by the tax surveyors (please see the Methods section for the detailed description of this taxation register and how it was used for building the dataset used in this study).

For several centuries before the 15th c. CE, the Peloponnese was predominantly inhabited by Greek-speaking populations engaged in mixed agriculture involving in particular grain and crop trees (1). The Albanians are first mentioned in the Peloponnese during the rule of Manuel Cantacuzenus in Mystras (1348–80). This mountain-dwelling transhumant population group that pursued pastoral economy migrated peacefully and in waves from Albania to Macedonia and Thessaly in the early 14th century. The Venetian and Byzantine authorities of the Peloponnese invited these wandering groups consisting of extended families or tribes to counterbalance the demographic losses of the Black Death and the almost constant warfare that ravaged the Moreot population in the second half of the 14th century (2-5); they offered them plots of arable land, pastures and tax exemptions. By 1391, there had been an influx of Albanians who could be hired as mercenaries (6-9). A well-attested-to more populous Albanian settlement took place in 1394–5 CE when ten thousand Albanians appeared before the Isthmus and asked Theodore I Palaeologus for permission to settle in the Peloponnese (10). A second wave of immigrants descended on the Morea perhaps in 1417–8 CE, most probably from southern Albania, after the Ottoman incursion, and western mainland Greece, where the Albanian rule was ended by Carlo Tocco (11-13).

At the first stage of their arrival, in the space of ten to thirty years, they were probably searching for appropriate land for animal husbandry; they soon, however, engaged themselves successfully in agriculture. The migration of Albanians during the late fourteenth century facilitated the establishment of Albanian population enclaves within the Peloponnese. These enclaves exhibited remarkable persistence, partially surviving into recent times, primarily due to their geographical isolation, which enabled the preservation of their distinct language, customs, and traditions (14-15). They gradually and peacefully occupied a vital territory, which was essential for the functioning of their economic and ecological system. This system necessitated vast and contiguous areas, suitable for the seasonal movements of these pastoralists whose ties to the land and ‘static’ activities were rather tenuous, particularly during the initial phase of their settlement (14).

Our first register - dated to 1460-63 CE - thus describes the situation in the Peloponnese 3-4 generations after the initial Albanian migration, which allows for the evaluation of the long-term role of culture in human niche construction: one may hypothesize that 3-4 generations was enough time for the migrants to give up ecological niche construction strategies they had used in their earlier environment and adapt for a new environment and the dominant mode of land use, represented by the Greek population (16).

The overall ecological differentiation into Greeks and newcomers of Albanian origin may have been related to different household formation, size and structure. Previous studies of populations living in the Ottoman-conquered Balkans, however, do not indicate that even in the late Middle Ages the various ethnic and religious groups must have been characterized by diametrically opposed demographic structures, or at least that ethnicity alone was not necessarily a differentiator (17-18). More important were local geographic and economic conditions (19). The sources we used defined farms “financially, in accordance with the taxpaying potential of its members rather than in accordance with its numerical size” (20), and we do not have detailed demographic data on the dominant family forms. We can guess that pastoralist communities were characterized by a relatively high incidence of households based on extended or joint families, which may also have been typical of agriculture oriented toward self-sufficiency. The commercialization of peacetime farming, a broader orientation toward market needs, and the development of land rights fostered the nucleation of households (21), better suited to the conditions of emerging proto-capitalism.

The preferential categorization of Albanians within the TT10–1/14662 (1460–63 CE) register was primarily driven by a 20% reduction in the poll tax burden (*ispence*), wherein they were assessed 20 *aḳçes* compared to the 25 *aḳçes* levied on Greeks. This fiscal distinction most likely reflects a late Byzantine and Venetian precedent subsequently adopted by the Ottomans, apparently as a means of managing the often resistant and rebellious Albanian population. Such a policy aligns with the broader Ottoman strategy of ‘continuity for stability’. However, this tax advantage is not evident in contemporary Ottoman registers for Trikala (1454/55 CE) and Euboea (1474 CE). Furthermore, mid-sixteenth-century law codes (*ḳānūnnāmes*) pertaining to the regions of Thebes and Livadeia explicitly detail taxes imposed on Albanian productive activities, yet these documents do not indicate any tax reductions for this group. Within approximately five decades, these favourable taxation terms for Albanians were rescinded. The TT80 register of the Peloponnese (1514/15 CE) indicates that both Greek and Albanian populations were subjected to the same annual *ispence* rate of 25 *aḳçes*, consequently eliminating the need to distinguish Albanian villages within the register. This standardisation suggests two key developments by the early sixteenth century: first, a consolidation of Ottoman authority across the peninsula, and second, the integration of the Albanian population into the local Greek societal structure (22).

**Supplementary Note S2**

**Taxation of vine and grain cultivation**

Wheat and wine were taxed at comparable rates, with taxes related to vineyard involving a more flexible and less predictable – potentially risky for taxpayers – system of estimating and collecting taxes. From the available evidence it is clear that the Ottoman state did everything it could to tax all aspects of vine cultivation and wine production, and thus to tap into this lucrative business and derive a substantial fiscal income from it. Consequently, in no way vine cultivation a tax-evasive activity compared to grain cultivation or animal herding.

More specifically, In the Ottoman fiscal system, apart from the poll tax paid in cash, the approach to agricultural tithe collection was contingent upon the specific crop. While the tithe on cereals and legumes was consistently rendered in kind – representing a direct portion of the harvest – tax collectors exercised discretion in determining the payment method for vineyard taxes, allowing for either in-kind or monetary remittance and often following pre-Ottoman practice. Viticulture, as a taxable activity, encompassed a range of levies that also extended to the process of vinification. Within the registers examined, these included taxes on vineyards and vineyard land (whether held by villagers or within the personal demesne of *timariots*), must, span (tax on must put in barrel for fermentation), wine, and raisins. The granular detail, localised differentiation, and comprehensive nature of this taxation system suggest a deliberate strategy designed to mitigate potential tax evasion within the viticultural sector (23). The preeminent role of viticulture in the calculation of monetary fief (*tīmār*) income, and consequently, in the imputed value of agricultural output, was directly correlated with wine prices (24).

While for grain the taxation was based on weight (a 10% in kind tax), for vine cultivation it was much more diversified, depending on the period and region of the empire, with the clear goal of raising as much tax as possible. The Ottoman administration employed a range of measurement units for assessing agricultural harvests and their corresponding tax liabilities, frequently aligning with established local practices for pragmatic reasons (25). In the case of cereals, assessment was based mostly on weight, with distinct standards for wheat compared to other grains. Additionally, the *ḥiml*, a unit of volume typically ranging from 6 to 8 *kile*, was also utilised for measurement of cereal production (26).

The comprehensive array and meticulous nature of viticulture imposts implemented during the Ottoman period are posited to have significantly constrained opportunities for tax evasion. Nevertheless, historical records indicate recurrent instances of wine smuggling, as attested by sultanic and judicial orders from the *mühimme defterleri* (decisions and decrees issued by the Imperial Council). In the case of our study region, the NW Peloponnese.

**Supplementary Note S3**

**The price of grain and wine per unit and their transport costs in Ottoman Greece**

Grain/flour and wine/must were two of the most frequently traded commodities in the Mediterranean, and the Ottoman Empire was no exception. When these goods reached town markets, wine/must commanded significantly higher prices than flour. For instance, in Edirne in 1502, the price of flour stood at 11 *aḳçe*s per *kile* of Istanbul, equivalent to 0.55 *aḳçe*s per *oka*, while must was priced ten times higher at 5.5 *aḳçe*s per *oka* (27) – the price difference was tenfold.

This price disparity can be partly attributed to two factors:

(a) Investment and labor: Viticulture requires substantial investment and demanding work (28-29).

(b) Freight costs: Transporting wine/must incurred higher costs compared to grain. For instance, in 1715, the sea transport cost from Istanbul or Tekirdağ to the Peloponnese was 5 *para*s per *oka* for wine or arak (equivalent to 3.9 *para*s per kg) and 15 *para*s per *kile* of Istanbul for grain (equivalent to 0.6 *para*s per kg) (30).

It can be surmised that similar differences in land freight costs between grain and wine existed in the 15^th^ and 16^th^-century Peloponnese. When land routes were chosen, grain was transported in sacks carried by animals. In contrast, wine/must was transported in wooden barrels — a dominant means of storage and transport in the Peloponnese from the 14^th^ century onward (29; 31) — on horses, mules, donkeys or carts (32-33). On the local level, according to the state legal codes, peasants were responsible for carrying at their own cost the grain tithe, which was collected in kind, to the nearest market (*aḳreb pazar*), provided that the distance did not exceed a day’s journey (34). The following examples shed light on land and sea freight costs: for a thirteen-day transport of grain for military purposes between Diyarbakır and Van in southeastern Anatolia in 1577, the freight cost amounted to 125% of the initial merchandise price, equivalent to 15 *aḳçe*s per *kile* of Istanbul. Whenever possible, cheaper maritime routes were preferred. For instance, a fifteen-day sea transport of grain from Alexandria to Istanbul in 1594 incurred a cost of 3 *aḳçe*s per *kile* of Istanbul (35).

**Supplementary Note S4**

**R code for checking for spatial autocorrelation using Moran’s test**

install.packages(c("spdep", "sf"))

library(spdep)

library(sf)

# Loading data

data <- read.csv("data.csv")

# Converting UTM coordinates to an sf object (spatial points). CRS for Hellenic Geodetic Reference System 1987 is 2100

sf_data <- st_as_sf(data, coords = c("coord_x", "coord_y"), crs = 2100)

for (k in c(5, 10, 15)) {

nb <- knn2nb(knearneigh(coords, k = k)) #nearest neighbors

listw <- nb2listw(nb, style = "W")

moran_test <- moran.test(sf_data$vineyards.tax.percent.cult.1460s, listw)

print(paste("k =", k, "1460s p-value =", moran_test$p.value, "Moran I 1460s = ", moran_test$estimate["Moran I statistic"]))

}

for (k in c(5, 10, 15)) {

nb <- knn2nb(knearneigh(coords, k = k)) #nearest neighbors

listw <- nb2listw(nb, style = "W")

moran_test <- moran.test(sf_data$vineyards.tax.percent.cult.1510s, listw)

print(paste("k =", k, "1510s p-value =", moran_test$p.value, "Moran I 1510s = ", moran_test$estimate["Moran I statistic"]))

}

for (k in c(5, 10, 15)) {

nb <- knn2nb(knearneigh(coords, k = k)) #nearest neighbors

listw <- nb2listw(nb, style = "W")

moran_test <- moran.test(sf_data$vineyards.tax.percent.cult.1580s, listw)

print(paste("k =", k, "1580s p-value =", moran_test$p.value, "Moran I 1580s = ", moran_test$estimate["Moran I statistic"]))

}

**Supplementary Note S5**

**Moran test results**

Not significant for 1460s

[1] "k = 5 1460s p-value = 0.159556180923353 Moran I 1460s = 0.0373540067341023"

[1] "k = 10 1460s p-value = 0.131895001585502 Moran I 1460s = 0.0286231806310605"

[1] "k = 15 1460s p-value = 0.153255049580588 Moran I 1460s = 0.0197752738850786"

Significant but weak for 1510s (threshold at c. 0.2)

[1] "k = 5 1510s p-value = 1.71568275590375e-10 Moran I 1510s = 0.264813548511101"

[1] "k = 10 1510s p-value = 1.52305663067248e-15 Moran I 1510s = 0.235596339662571"

[1] "k = 15 1510s p-value = 5.92564891426713e-19 Moran I 1510s = 0.212514121563322"

Not significant for 1580s

[1] "k = 5 1580s p-value = 0.49438446296019 Moran I 1580s = -0.00486183028798253"

[1] "k = 10 1580s p-value = 0.158732430674833 Moran I 1580s = 0.0249123468363581"

[1] "k = 15 1580s p-value = 0.0488725356760881 Moran I 1580s = 0.0352495785734327"

**Figure S1.** Reconstructed 600-1200 CE June-July-August hydroclimate (PDSI) for southern Greece (Peloponnese), based on the Old World Drought Atlas (<http://drought.memphis.edu>) (36).


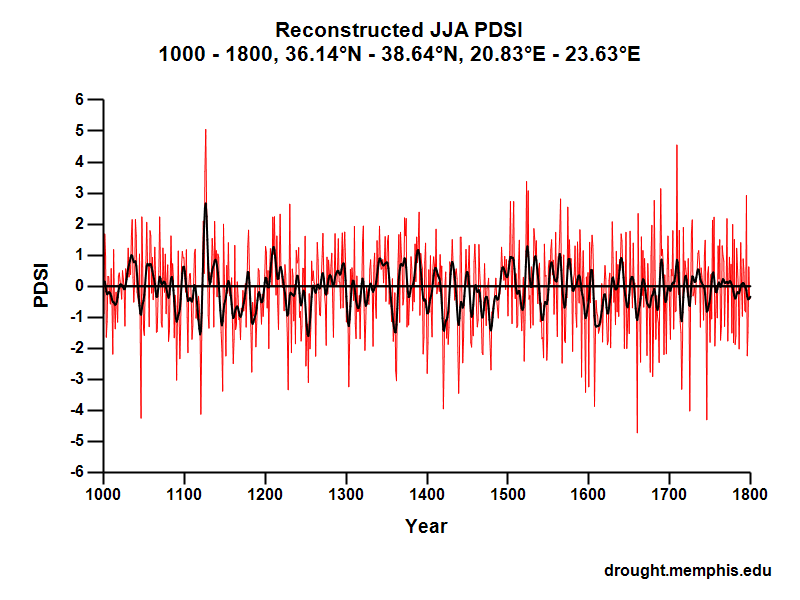


**Figure S2.** regional distribution of average household agricultural tax at village level (reflecting villages affluence). Figure elements represent the median line (middle of the box), the first and third quartiles (box), average (x), and outliers (individual points).


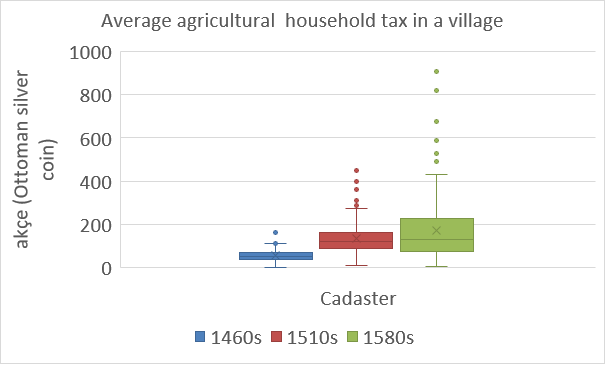


**Figure S3.** Comparison of observed vs. model predicted (best BIC) values for the 1460s time bin. 6% of predictions are below 0%. None are above 100%.


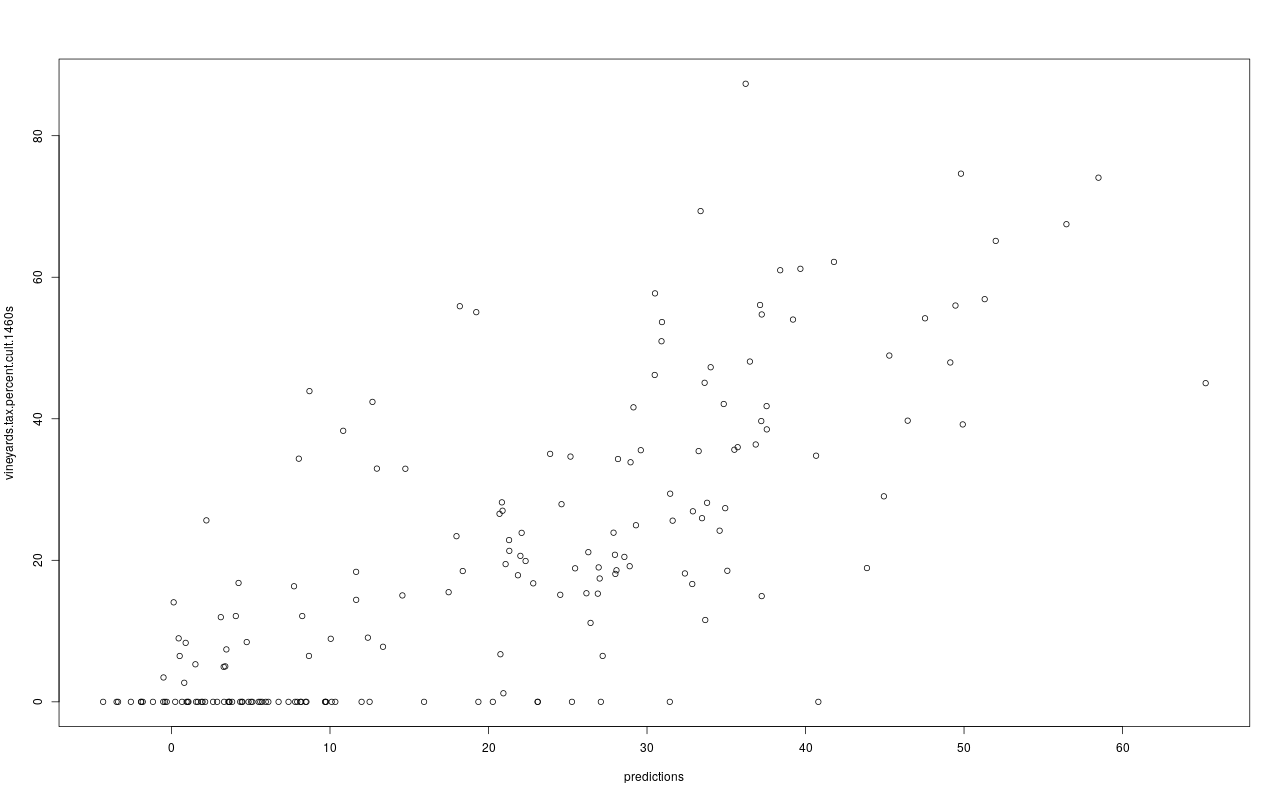


**Figure S4.** Comparison of observed vs. model predicted (best BIC) values for the 1510s time bin. No predictions are outside the 0 to 100% range.


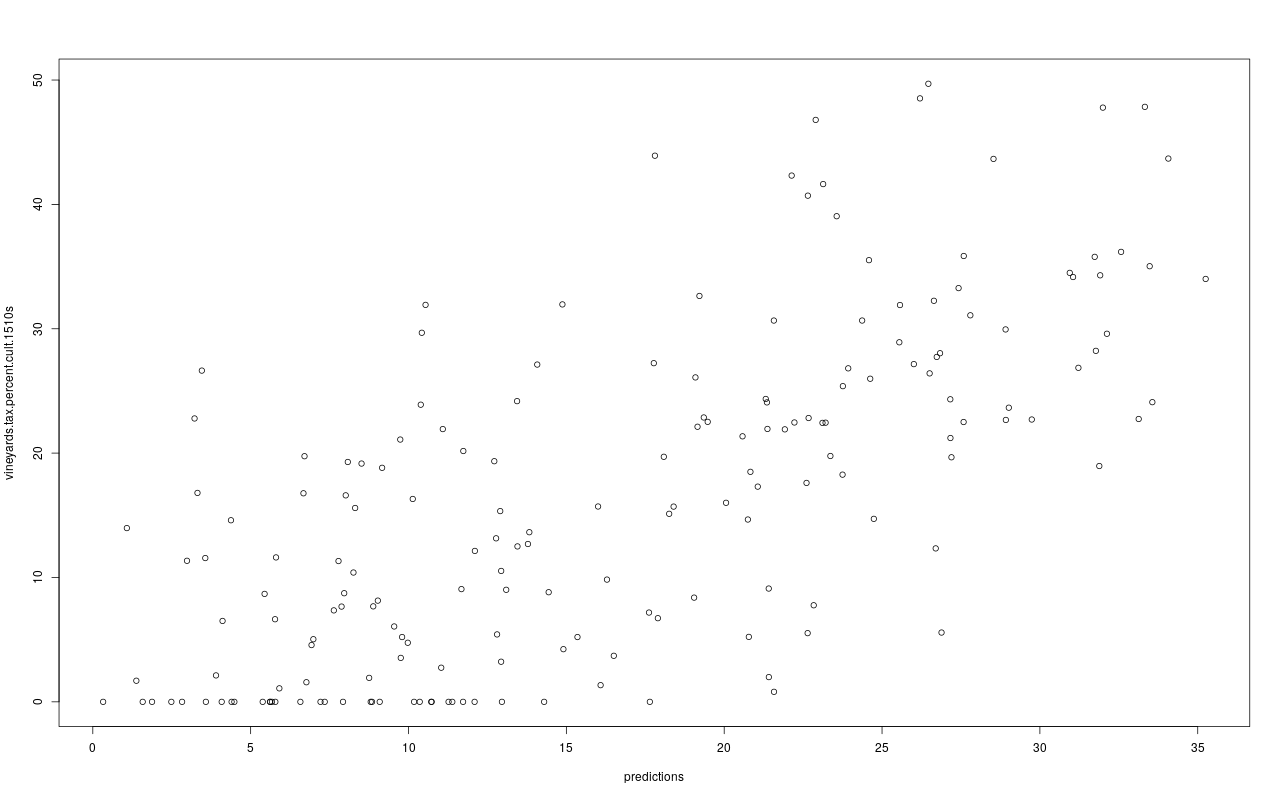


**Figure S5.** Comparison of observed vs. model predicted (best BIC) values for the 1580s time bin. No predictions are outside the 0 to 100% range.


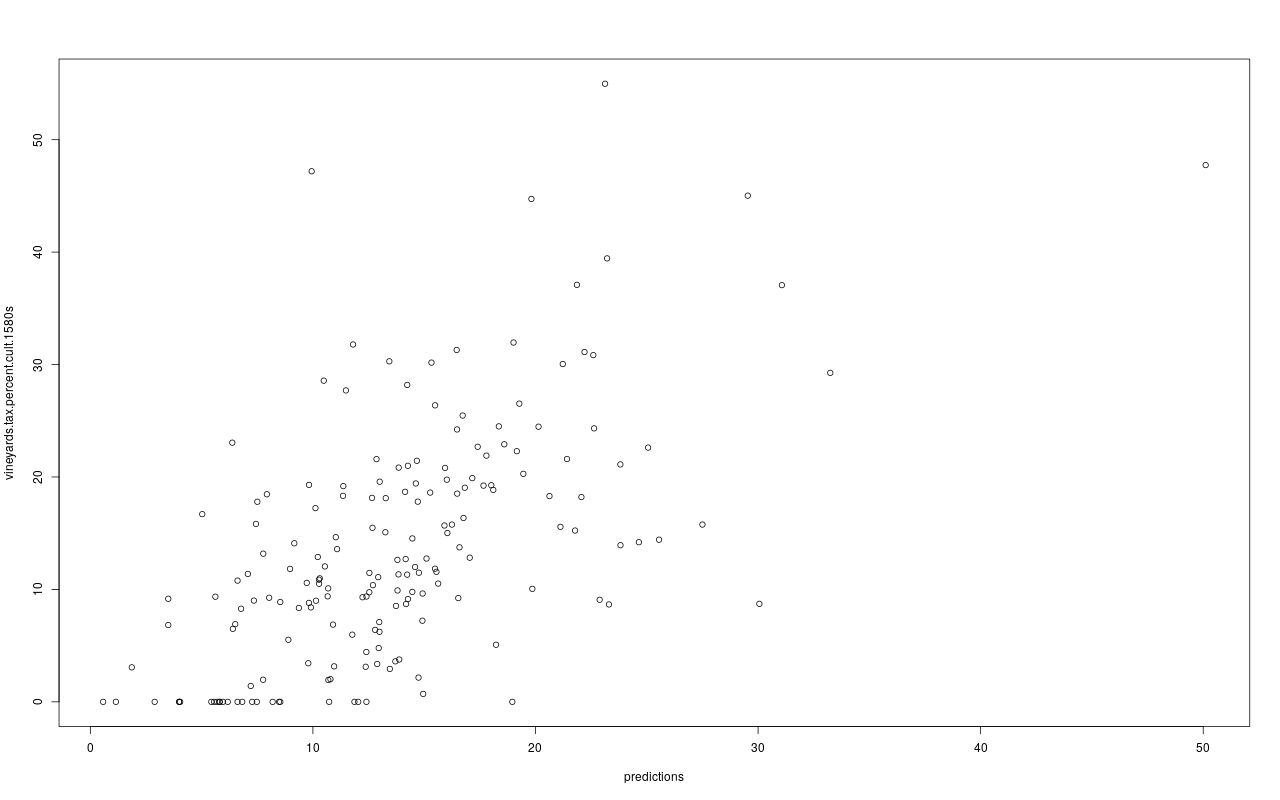


**Table S1.** Bayesian Information Criterion (BIC) and Bayesian R^2^ values. The optimal model for each register in bold (BIC-based).

| Model | Bayes_Rsq | rank | BIC | rank |
| --- | --- | --- | --- | --- |
| **1460s** | | | | |
| Ethnicity | 0,42254773 | 8 | 432,743097 | 8 |
| ethnicity + taxavg.1460s | 0,55256355 | 7 | 391,589181 | 2 |
| **ethnicity + taxavg.1460s + river.distance** | **0,57634897** | **5** | **387,617396** | **1** |
| ethnicity + taxavg.1460s + river.distance + taxavg.1460s^2 | 0,57603953 | 6 | 393,821176 | 3 |
| ethnicity + taxavg.1460s + river.distance + taxavg.1460s^2 + households.1460s:taxavg.1460s^2 | 0,58542939 | 4 | 395,687922 | 4 |
| ethnicity + taxavg.1460s + river.distance + taxavg.1460s^2 + households.1460s:taxavg.1460s^2 + households.1460s^2:taxavg.1460s^2 | 0,5856783 | 3 | 401,679209 | 5 |
| ethnicity + taxavg.1460s + river.distance + taxavg.1460s^2 + households.1460s:taxavg.1460s^2 + households.1460s^2:taxavg.1460s^2 + households.1460s:taxavg.1460s | 0,58774588 | 2 | 406,798717 | 6 |
| ethnicity + taxavg.1460s + river.distance + taxavg.1460s^2 + households.1460s:taxavg.1460s^2 + households.1460s^2:taxavg.1460s^2 + households.1460s:taxavg.1460s + river.distance^2 | 0,58760277 | 1 | 412,881512 | 7 |
| **1510s** | | | | |
| ethnicity | 0,32235193 | 8 | 462,383637 | 8 |
| ethnicity + households.1510s | 0,36802838 | 7 | 456,055208 | 4 |
| ethnicity + households.1510s + households.1510s:taxavg.1510s | 0,36936298 | 6 | 462,175281 | 7 |
| ethnicity + households.1510s + households.1510s:taxavg.1510s + HARBOUR.distance | 0,42082865 | 5 | 452,488819 | 2 |
| ethnicity + households.1510s + households.1510s:taxavg.1510s + HARBOUR.distance + altitude | 0,42500474 | 4 | 457,476498 | 6 |
| ethnicity + households.1510s + households.1510s:taxavg.1510s + HARBOUR.distance + altitude + households.1510s^2 | 0,44795529 | 3 | 456,005933 | 3 |
| **ethnicity + households.1510s + households.1510s:taxavg.1510s + HARBOUR.distance + altitude + households.1510s^2 + altitude^2** | **0,47821197** | **2** | **451,580862** | **1** |
| ethnicity + households.1510s + households.1510s:taxavg.1510s + HARBOUR.distance + altitude + households.1510s^2 + altitude^2 + taxavg.1510s:households.1510s^2 | 0,47982208 | 1 | 457,33349 | 5 |
| **1580s** | | | | |
| households.1580s:taxavg.1580s | 0,23164476 | 8 | 485,907783 | 7 |
| households.1580s:taxavg.1580s + taxavg.1580s:households.1580s^2 | 0,2760401 | 7 | 481,392208 | 4 |
| households.1580s:taxavg.1580s + taxavg.1580s:households.1580s^2 + ethnicity | 0,31381482 | 6 | 478,016091 | 2 |
| households.1580s:taxavg.1580s + taxavg.1580s:households.1580s^2 + ethnicity + altitude^2 | 0,31801816 | 5 | 483,472952 | 5 |
| **households.1580s:taxavg.1580s + taxavg.1580s:households.1580s^2 + ethnicity + altitude^2 + altitude** | **0,37025971** | **4** | **474,844871** | **1** |
| households.1580s:taxavg.1580s + taxavg.1580s:households.1580s^2 + ethnicity + altitude^2 + altitude + households.1580s^2:taxavg.1580s^2 | 0,37239809 | 3 | 480,502258 | 3 |
| households.1580s:taxavg.1580s + taxavg.1580s:households.1580s^2 + ethnicity + altitude^2 + altitude + households.1580s^2:taxavg.1580s^2 + HARBOUR.distance^2 | 0,37964481 | 2 | 484,846349 | 6 |
| households.1580s:taxavg.1580s + taxavg.1580s:households.1580s^2 + ethnicity + altitude^2 + altitude + households.1580s^2:taxavg.1580s^2 + HARBOUR.distance^2 + households.1580s^2 | 0,38140982 | 1 | 490,837622 | 8 |

**Table S2.** Correlations between the independent variables (numerical; additional the eta value for altitude [numerical] and soils [categorical]). Correlations above 0.7 in bold.

|  | river.distance | precip.winter | precip.spring | precip.summer | precip.autumn | avg.temp.winter | avg.temp.spring | avg.temp.summer | avg.temp.autumn | slope.median | altitude | CAPITAL.distance | HARBOR.distance |
| --- | --- | --- | --- | --- | --- | --- | --- | --- | --- | --- | --- | --- | --- |
| river.distance | 1 | -0.120281686717736 | -0.0446451566634876 | -0.0429293374427584 | -0.0563771226733498 | 0.0194292117617077 | 0.0342538533289818 | 0.0355854672611363 | 0.052554943099 | -0.0560972400646459 | -0.0369691868949007 | -0.0423361637218484 | -0.0101665376531538 |
| precip.winter | -0.120281686717736 | 1 | **0.862348993219236** | 0.599819876740416 | 0.660556502133662 | **-0.753425201713073** | **-0.750008650175125** | -0.691649196134999 | **-0.719356508831841** | 0.616405725440071 | 0.687235802625125 | 0.233198658677013 | 0.346978835762138 |
| precip.spring | -0.0446451566634876 | **0.862348993219236** | 1 | **0.810899547363712** | **0.703816595563662** | **-0.887055903090003** | **-0.876427056838685** | **-0.83132147179259** | **-0.860172471982334** | **0.79481798421335** | **0.840858582960487** | 0.188492492674517 | 0.462508321518871 |
| precip.summer | -0.0429293374427584 | 0.599819876740416 | **0.810899547363712** | 1 | 0.425763617162968 | **-0.813450426864212** | **-0.795906849788514** | **-0.769098431806074** | **-0.792206741832103** | **0.747296358015939** | **0.804641068441096** | 0.216094515207372 | 0.614224594523849 |
| precip.autumn | -0.0563771226733498 | 0.660556502133662 | **0.703816595563662** | 0.425763617162968 | 1 | -0.381177577875311 | -0.369867321519394 | -0.321370556311374 | -0.337675439583161 | 0.244154503179862 | 0.316367994540648 | 0.125018457610236 | 0.23555221752771 |
| avg.temp.winter | 0.0194292117617077 | **-0.753425201713073** | **-0.887055903090003** | **-0.813450426864212** | -0.381177577875311 | 1 | **0.990627664452526** | **0.961721987706872** | **0.986879684182391** | -0.870292774559869 | **-0.964295501731818** | -0.232730993506817 | -0.479543230147028 |
| avg.temp.spring | 0.0342538533289818 | **-0.750008650175125** | **-0.876427056838685** | **-0.795906849788514** | -0.369867321519394 | **0.990627664452526** | 1 | **0.986776281349273** | **0.994293584454093** | **-0.863498950841131** | **-0.972651903286793** | -0.202498326099298 | -0.43490500970849 |
| avg.temp.summer | 0.0355854672611363 | -0.691649196134999 | **-0.83132147179259** | **-0.769098431806074** | -0.321370556311374 | **0.961721987706872** | **0.986776281349273** | 1 | **0.981608665780328** | **-0.841659040848519** | **-0.965963571069411** | -0.158815560659534 | -0.406608417172108 |
| avg.temp.autumn | 0.052554943099 | **-0.719356508831841** | **-0.860172471982334** | **-0.792206741832103** | -0.337675439583161 | **0.986879684182391** | **0.994293584454093** | **0.981608665780328** | 1 | **-0.881117309653146** | **-0.976199721691178** | -0.192742085006384 | -0.437788222098014 |
| altitude | -0.0369691868949007 | 0.687235802625125 | **0.840858582960487** | **0.804641068441096** | 0.316367994540648 | **-0.964295501731818** | **-0.972651903286793** | **-0.965963571069411** | **-0.976199721691178** | **0.857547692628501** | 1 | 0.163261139511123 | 0.422680491111028 |
| CAPITAL.distance | -0.0423361637218484 | 0.233198658677013 | 0.188492492674517 | 0.216094515207372 | 0.125018457610236 | -0.232730993506817 | -0.202498326099298 | -0.158815560659534 | -0.192742085006384 | 0.174070507684283 | 0.163261139511123 | 1 | 0.463604792749535 |
| HARBOR.distance | -0.0101665376531538 | 0.346978835762138 | 0.462508321518871 | 0.614224594523849 | 0.23555221752771 | -0.479543230147028 | -0.43490500970849 | -0.406608417172108 | -0.437788222098014 | 0.46892135656578 | 0.422680491111028 | 0.463604792749535 | 1 |
| slope.median | -0.0560972400646459 | 0.616405725440071 | **0.79481798421335** | **0.747296358015939** | 0.244154503179862 | **-0.870292774559869** | **-0.863498950841131** | **-0.841659040848519** | **-0.881117309653146** | 1 | **0.857547692628501** | 0.174070507684283 | 0.46892135656578 |
| Eta soils (categorical) : altitude: **0.7531** | | | | | | | | | | | | | |

**Table S3.** Model diagnostics for models presented in Table 3.

|  | Gelman's PSRF | | Raftery and Lewis | | | | Geweke | Heidelberg and Welch | | | | | |
| --- | --- | --- | --- | --- | --- | --- | --- | --- | --- | --- | --- | --- | --- |
|  | Point est. | Upper C.I. | Burn-in (M) | Total (N) | Lower bound (Nmin) | Depen-dence factor (I) | z-score | Station-arity test | start itera-tion | p-value | Half-width test | Mean | Halfwidth |
| 1460s | | | | | | | | | | | | | |
| (Intercept) | 1 | 1 | 2 | 3736 | 3746 | 0.997 | 1.24791 | passed | 1 | 0.646 | failed | -0.000336 | 0.000338 |
| ethnicityGR | 1 | 1 | 2 | 3783 | 3746 | 1.010 | -0.76338 | passed | 1 | 0.297 | passed | 0.514131 | 0.000361 |
| taxavg.1460s | 1 | 1 | 2 | 3740 | 3746 | 0.988 | -0.02256 | passed | 1 | 0.922 | passed | 0.378646 | 0.000362 |
| river.distance | 1 | 1 | 1 | 3743 | 3746 | 0.999 | -0.56186 | passed | 1 | 0.818 | passed | 0.155202 | 0.000338 |
| 1510s | | | | | | | | | | | | | |
| (Intercept) | 1 | 1 | 2 | 3736 | 3746 | 0.997 | 0.05972 | passed | 1 | 0.0713 | failed | 0.00018 | 0. 000379 |
| ethnicityGR | 1 | 1 | 1 | 3747 | 3746 | 1.000 | 0.37233 | passed | 1 | 0.6399 | passed | 0.27763 | 0.000488 |
| households.1510s | 1 | 1 | 2 | 3763 | 3746 | 1.000 | -0. 30744 | passed | 1 | 0.8398 | passed | 0.66685 | 0.001368 |
| HARBOUR.distance | 1 | 1 | 1 | 3755 | 3746 | 1.000 | 0. 33579 | passed | 8001 | 0.2123 | passed | 0.19473 | 0.000452 |
| altitude | 1 | 1 | 2 | 3783 | 3746 | 1.010 | -0. 05487 | passed | 1 | 0.4840 | passed | 0.72291 | 0.001486 |
| I(households.1510s^2) | 1 | 1 | 2 | 3736 | 3746 | 0.977 | 0. 08961 | passed | 1 | 0.4964 | passed | -0.47706 | 0.001060 |
| I(altitude^2) | 1 | 1 | 1 | 3747 | 3746 | 1.000 | 0. 09552 | passed | 1 | 0.6061 | passed | -0.67471 | 0.001446 |
| households.1510s:taxavg.1510s | 1 | 1 | 2 | 3736 | 3746 | 0.997 | 0. 40376 | passed | 1 | 0.6739 | passed | 0.05573 | 0.000743 |
| 1580s | | | | | | | | | | | | | |
| (Intercept) | 1 | 1 | 2 | 3717 | 3746 | 0.992 | -0.27637 | passed | 1 | 0.741 | failed | 0.0002 | 0.000418 |
| ethnicityGR | 1 | 1 | 2 | 3787 | 3746 | 1.010 | -0.15306 | passed | 1 | 0.642 | passed | 0.1393 | 0.000468 |
| I(altitude^2) | 1 | 1 | 2 | 3740 | 3746 | 0.998 | -0.10982 | passed | 1 | 0.479 | passed | -0.8945 | 0.001569 |
| altitude | 1 | 1 | 2 | 3698 | 3746 | 0.987 | -0.02551 | passed | 1 | 0.429 | passed | 0.8877 | 0.001597 |
| taxavg.1580s:households.1580s | 1 | 1 | 2 | 3736 | 3746 | 0.997 | -0.42958 | passed | 1 | 0.235 | passed | 0.8536 | 0.001027 |
| taxavg.1580s:I(households.1580s^2) | 1 | 1 | 2 | 3760 | 3746 | 1.000 | 0.40548 | passed | 1 | 0.307 | passed | -0.4536 | 0.000998 |

**SI References**

(1) K. D. Papakosma, *Η αγροτική ζωή στην Πελοπόννησο κατά την ύστερη βυζαντινή εποχή (κοινωνικο-οικονομικά στοιχεία για τους 13ο-150 αι.)*, (National and Kapodistrian University of Athens, 2010).

(2) D. A. Zakythinos, La population de la Morée byzantine. *L’Hellénisme contemporain* **3/1**, 9–10 (1949).

(3) V. Panagiotopoulos, *Πληθυσμός και οικισμοί της Πελοποννήσου, 13^ος^-18^ος^ αιώνας*, Μελέτες Νεοελληνικής Ιστορίας (Ιστορικό Αρχείο, Εμπορική Τράπεζα της Ελλάδος, 1987), pp. 61–68.

(4) Th. E. Detorakis, “Ἐνθυμητικαὶ καὶ ἱστορικαὶ μαρτυρίαι περὶ ἐπιδημιῶν πανώλους εἰς Πελοπόννησον” in *Πρακτικὰ τοῦ Α΄ Διεθνοῦς Συνεδρίου Πελοποννησιακῶν Σπουδῶν (Σπάρτη, 7-14 Σεπτεμβρίου 1975)*, vol. 3, Πελοποννησιακὰ Παράρτημα 6 (Ἑταιρεία Πελοποννησιακῶν Σπουδῶν, 1976-1978), pp. 15-21.

(5) K. P. Kostis, *Στον καιρό της πανώλης. Εικόνες από τις κοινωνίες της ελληνικής χερσονήσου. 14^ος^-19^ος^ αιώνας* (Πανεπιστημιακές Εκδόσεις Κρήτης, 2020), pp. 303–320.

(6) F. Thiriet, *La Romanie vénitienne au moyen âge. Le développement et l’exploitation du domaine colonial vénitien (XII^e^-XV^e^ siècles)* (De Boccard, 1959), p. 366.

(7) J. Chrysostomides, *Monumenta Peloponnesiaca. Documents for the History of the Peloponnese in the 14^th^ and 15^th^ Centuries* (Porphyrogenitus, 1995), pp. 206, 291, 337, 339.

(8) P. Topping, “Albanian Settlements in Medieval Greece: Some Venetian Testimonies” in *Charanis Studies. Essays in Honor of Peter Charanis*. A. E. Laiou-Thomadakis, Ed. (Rutgers University Press, 1980), pp. 261-271.

(9) A. Ducellier, Les Albanais dans les colonies vénitiennes au XV^e^ siècle. *Studi Veneziani* **10**, 47-64 (1968).

(10) M. Palaeologus, *Funeral Oration on his Brother Theodore*, Introduction, text, translation and notes by J. Chrysostomides, Corpus Fontium Historiae Byzantinae 26, Series Thessalonicensis (Association for Byzantine Research, 1985), p. 120.

(11) A. Ducellier, *Οι Αλβανοί στην Ελλάδα (13^ος^-15^ος^ αι.). Η μετανάστευση μιας κοινότητας*, K. Nikolaou, Trans., Όψεις της Βυζαντινής Κοινωνίας 15 (Ίδρυμα Γουλανδρή Χορν, 2000), p. 40.

(12) I. H. Poulos, Ἡ ἐποίκησις τῶν Ἀλβανῶν εἰς Κορινθίαν. *Ἐπετηρὶς τοῦ Μεσαιωνικοῦ Ἀρχείου* **3**, 70–86 (1950).

(13) J. G. von Hahn, *Albanesische Studien. 1. Heft. Geographisch-ethnographische Uebersicht. Reiseskizzen. Sittenschilderungen. Sind die Albanesen Autochthonen? Das albanische Alphabet. Historisches* (Verlag von Friedrich Mauke, Druck der kaiserlich-königlichen Hof- und Staatsdruckerei in Wien, 1854), p. 322.

(14) Panagiotopoulos, V. Πληθυσμός και οικισμοί της Πελοποννήσου, 13^ος^–18^ος^ αιώνας. *Μελέτες Νεοελληνικής Ιστορίας* (Ιστορικό Αρχείο, Εμπορική Τράπεζα της Ελλάδας, 1987).

(15) Giohalas, T. Η αρβανιτιά στο Μοριά. Χρονικά πορείας, 2 vols (Πατάκη, 2011).

(16) G. C. Liakopoulos, “The Integration of Settlers into Existing Socio-Environmental Settings: Reclaiming the Greek Lands After the Late Medieval Crisis” in *Perspectives on Public Policy in Societal-Environmental Crises. Risk, Systems and Decisions*, A. Izdebski, J. Haldon, P. Filipkowski, Eds. (Springer, 2022), pp. 307-324.

(17) E. A. Hammel, Sensitivity analysis of household structure in medieval Serbian censuses. *Historical Methods: A Journal of Quantitative and Interdisciplinary History* **13(2)**, 105-118 (1980).

(18) M. Bobić, “The Reconstruction of Domestic Communities in the Branković Region of Serbia in 1455” in *The History of Families and Households: Comparative European Dimensions* (Brill, 2018), pp. 69-92.

(19) M. Mitterauer, Family contexts: the Balkans in European comparison. *The History of the Family*, **1(4)**, 387-406 (1996).

(20) K. H. Karpat, *Ottoman Population 1830-1914. Demographic and Social Characteristics* (University of Wisconsin Press, 1985).

(21) M. Mitterauer, Peasant and non-peasant family forms in relation to the physical environment and the local economy. *Journal of Family History*, **17(2)**, 139-159 (1992).

(22) Liakopoulos, G. C. The early Ottoman Peloponnese: A study in the light of an annotated editio princeps of the TT10-1/14662 Ottoman taxation cadastre (ca. 1460-1463). *Royal Asiatic Society, Ibrahim Pasha of Egypt Series* (Gingko, 2019).

(23) Kabrda, J. Contribution à l'étude de la rente féodale dans l'empire Ottoman I : (redevances féodales perçues sur le vin et le miel). *Sborník prací Filozofické fakulty brněnské univerzity. C, Řada historická* **12/C10** (1963), pp. 34–39.

(24) Asdrachas, S. Ι. Η αγροτική οικονομία. In Ελληνική οικονομική ιστορία ΙΕ΄-ΙΘ΄ αιώνας (ed. Asdrachas, S. I.) vol. 1, p. 296 (Πολιτιστικό Ίδρυμα Ομίλου Πειραιώς, 2003).

(25) İnalcık, H. Introduction to Ottoman Metrology. *Turcica* **15** (1983), p. 312.

(26) Taşkın, Ü. Osmanlı Devleti’nde Kullanılan Ölçü ve Tartı Birimleri. MA dissertation (T.C. Fırat Üniversitesi, 2005), pp. 119–123.

(27) N. Beldiceanu, *Recherche sur la ville ottomane au XVe siècle. Étude et actes*, Bibliothèque archéologique et historique de l’Institut Français d'archéologie d’Istanbul 25 (Librairie d’Amérique et d’Orient, Adrien Maisonneuve, 1973), pp. 252–253, 255.

(28) P. Topping, “Viticulture in Venetian Crete (XIIIth c.)” in *Πεπραγμένα του Δ΄ Διεθνούς Κρητολογικού Συνεδρίου (Ηράκλειο, 29 Αυγούστου - 3 Σεπτεμβρίου 1976). Τόμος Β΄ Βυζαντινοί και Μέσοι Χρόνοι* (Πανεπιστήμιο Κρήτης, 1981), pp. 515, 518.

(29) A. Panopoulou, K. Papakosma, “Vinee and vinum. Αμπελοκαλλιέργεια και παραγωγή κρασιού στη δυτική Πελοπόννησο (13ος-15ος αι.)” in *Επιστημονικό Συμπόσιο Οἶνον Ἱστορῶ. Αμπελοοινική ιστορία και αρχαιολογία της ΒΔ Πελοποννήσου*, Y. A. Pikoulas, Ed. (Κτήμα Μερκούρη, Κορακοχώρι Ηλείας, 2001), p. 120.

(30) K. Şakul, Military Transportation as Part of Mediterranean Maritime Trade: Ottoman Freight Payments during the War of the Second Coalition (1798-1802). *Journal of Mediterranean Studies* **19/2**, 396 (2010).

(31) I. Anagnostakis, “Βυζαντινά οινοβούτια, βουτζία και Βουτζαράδες του Αρακλόβου στην φραγκοκρατούμενη Ηλεία”, in *Επιστημονικό Συμπόσιο Οἶνον Ἱστορῶ. Αμπελοοινική ιστορία και αρχαιολογία της ΒΔ Πελοποννήσου*, Y. A. Pikoulas, Ed. (Κτήμα Μερκούρη, Κορακοχώρι Ηλείας, 2001), pp. 99–100.

(32) K. G. Tsiknakis, Ed., *Il miglior vino del mondo. Το κρητικό κρασί στις αρχειακές πηγές της βενετοκρατίας* (Δήμος Γαζίου, 2005), p. 104.

(33) V. Kremmydas, *Τὸ ἐμπόριο τῆς Πελοποννήσου στὸ 18^ο^ αἰώνα (1715–1792) (μὲ βάση τὰ γαλλικὰ ἀρχεῖα)* (Τυπογραφία Φ. Κωνσταντινίδη καὶ Κ. Μιχαλᾶ Ο.Ε., 1972), p. 258.

(34) H. İslamoğlu-İnan, “State and Peasants in the Ottoman Empire: A Study of Peasant Economy in North-central Anatolia During the Sixteenth Century” in *The Ottoman Empire and the World-Economy*, H. İslamoğlu-İnan, Ed., Studies in Modern Capitalism. Études sur le capitalisme moderne (Cambridge University Press, Éditions de la Maison des Sciences de l'Homme, 1987), p. 104.

(35) L. Güçer, *XVI-XVII. Asırlarda Osmanlı İmparatorluğunda Hububat Meselesi ve Hububattan Alınan Vergiler*, İstanbul Üniversitesi Yayınlarından İktisat Fakültesi 1075/152 (Sermet Matbaası, Şemsi Arkadaş, 1964), pp. 28–29, 33.

(36) E.R. Cook *et al.*, Old World megadroughts and pluvials during the Common Era. *Science Advances* **1/10** (2015), doi:10.1126/sciadv.1500561.
