# Supplementary material for: Emerging capitalism and sociocultural niche construction in early modern Greece
Source: Nat Commun. 2026 Jul 22;17:7119. doi: 10.1038/s41467-026-75315-y (PMC13396767; doi:10.1038/s41467-026-75315-y)
Supplement: Supplementary file 2 — Reporting Summary [file 41467_2026_75315_MOESM2_ESM.pdf]

nature portfolio

Corresponding author(s): Liakopoulos

Last updated by author(s): Jan 16, 2026

Reporting Summary

Nature Portfolio wishes to improve the reproducibility of the work that we publish. This form provides structure for consistency and transparency in reporting. For further information on Nature Portfolio policies, see our Editorial Policies and the Editorial Policy Checklist.

Statistics

For all statistical analyses, confirm that the following items are present in the figure legend, table legend, main text, or Methods section.

n/a	Confirmed	
☐	☒	The exact sample size (n) for each experimental group/condition, given as a discrete number and unit of measurement
☒	☐	A statement on whether measurements were taken from distinct samples or whether the same sample was measured repeatedly
☐	☒	The statistical test(s) used AND whether they are one- or two-sided<br>*Only common tests should be described solely by name; describe more complex techniques in the Methods section.*
☐	☒	A description of all covariates tested
☒	☐	A description of any assumptions or corrections, such as tests of normality and adjustment for multiple comparisons
☐	☒	A full description of the statistical parameters including central tendency (e.g. means) or other basic estimates (e.g. regression coefficient) AND variation (e.g. standard deviation) or associated estimates of uncertainty (e.g. confidence intervals)
☐	☒	For null hypothesis testing, the test statistic (e.g. F, t, r) with confidence intervals, effect sizes, degrees of freedom and P value noted<br>*Give P values as exact values whenever suitable.*
☐	☒	For Bayesian analysis, information on the choice of priors and Markov chain Monte Carlo settings
☒	☐	For hierarchical and complex designs, identification of the appropriate level for tests and full reporting of outcomes
☒	☐	Estimates of effect sizes (e.g. Cohen's d, Pearson's r), indicating how they were calculated

Our web collection on statistics for biologists contains articles on many of the points above.

Software and code

Policy information about availability of computer code

Data collection	No code was used for historical data collection; GIS data generation procedure is described in the methods.
Data analysis	The full R-code for BMSC, including access to a corresponding online app, is available at the Pandora & IsoMemo software repository (https://github.com/Pandora-IsoMemo/bmsc-app).

For manuscripts utilizing custom algorithms or software that are central to the research but not yet described in published literature, software must be made available to editors and reviewers. We strongly encourage code deposition in a community repository (e.g. GitHub). See the Nature Portfolio guidelines for submitting code & software for further information.

Data

Policy information about availability of data

All manuscripts must include a data availability statement. This statement should provide the following information, where applicable:

- Accession codes, unique identifiers, or web links for publicly available datasets
- A description of any restrictions on data availability
- For clinical datasets or third party data, please ensure that the statement adheres to our policy

The underlying dataset is available on the Pandora data platform via the “Environmental History” data community and the “Tax records for Ottoman Peloponnese” repository: https://www.doi.org/10.48493/rz8t-wv22.

Research involving human participants, their data, or biological material

Policy information about studies with human participants or human data. See also policy information about sex, gender (identity/presentation), and sexual orientation and race, ethnicity and racism.

Reporting on sex and gender	n/a
Reporting on race, ethnicity, or other socially relevant groupings	We use the term ethnicity to differentiate between the different cultural-linguistic idenitites of Greek and Albanian speakers in medieval and early modern Greece, in line with the historical sources.
Population characteristics	n/a
Recruitment	n/a
Ethics oversight	n/a

Note that full information on the approval of the study protocol must also be provided in the manuscript.

Field-specific reporting

Please select the one below that is the best fit for your research. If you are not sure, read the appropriate sections before making your selection.

☐ Life sciences ☐ Behavioural & social sciences ☒ Ecological, evolutionary & environmental sciences

For a reference copy of the document with all sections, see nature.com/documents/nr-reporting-summary-flat.pdf

Ecological, evolutionary & environmental sciences study design

All studies must disclose on these points even when the disclosure is negative.

Study description	Machine-aided Bayesian regression exploration of historical and modern envionrmental data.
Research sample	All villages recorded in the Ottoman taxation cadaster of the Province of Morea in the 1460s.
Sampling strategy	No sampling procedure - we included all villages meeting our criteria.
Data collection	The data were sampled by Georgios Liakopolus and Dimitrios Lamprakis from published or archival copies of Ottoman taxation registers. Georgios Liakopoulos geolocalized the villages. Theodoros Vakkas used GIS approaches to collect geogrpahical (environmental) data.
Timing and spatial scale	n/a
Data exclusions	n/a
Reproducibility	We provide the full datastet which makes it possible to reproduce our analysis. It is also possible to reproduce data collection, either by applying the GIS procedures described in the paper or accessing the manuscripts and extracting the data on one's own (which took us 3 years, however).
Randomization	n/a
Blinding	n/a

Did the study involve field work? ☐ Yes ☒ No

Reporting for specific materials, systems and methods

We require information from authors about some types of materials, experimental systems and methods used in many studies. Here, indicate whether each material, system or method listed is relevant to your study. If you are not sure if a list item applies to your research, read the appropriate section before selecting a response.

Materials & experimental systems

n/a	Involved in the study	
☒	☐	Antibodies
☒	☐	Eukaryotic cell lines
☒	☐	Palaeontology and archaeology
☒	☐	Animals and other organisms
☒	☐	Clinical data
☒	☐	Dual use research of concern
☒	☐	Plants

Methods

n/a	Involved in the study	
☒	☐	ChIP-seq
☒	☐	Flow cytometry
☒	☐	MRI-based neuroimaging

Plants

Seed stocks	n/a
Novel plant genotypes	n/a
Authentication	n/a
