# Supplementary material for: Emerging capitalism and sociocultural niche construction in early modern Greece
Source: Nat Commun. 2026 Jul 22;17:7119. doi: 10.1038/s41467-026-75315-y (PMC13396767; doi:10.1038/s41467-026-75315-y)
Supplement: Supplementary file 3 — Transparent Peer Review file [file 41467_2026_75315_MOESM3_ESM.pdf]

nature portfolio

Peer Review File

Emerging capitalism and sociocultural niche construction in early modern Greece

Corresponding Author: Dr Georgios Liakopoulos

This file contains all reviewer reports in order by version, followed by all author rebuttals in order by version.

Version 0:

Reviewer comments:

Reviewer #1

(Remarks to the Author)
This paper uses data from three tax registers of the Ottoman Empire from the15th-16th centuries to study human niche construction in a historical context, specifically to compare the evolution of land use choices of Albanian and Greek village communities in southern Greece over time. To estimate the effect of ethnicity, the authors apply a Bayesian machine learning algorithm to a geospatial quantitative dataset. They find that under Ottoman rule these communities experienced a significant economic transition. Whereas Greek/Albanian ethno-cultural identities previously played a major role in determining local niche construction strategies, successive generations under Ottoman rule shifted to strategies based on the environmental characteristics of their surroundings and the investment potential of each village community. The authors interpret this phenomenon as being a product of the transition from unstable political conditions and limited commodification of agriculture to lasting peace, intense market integration and strong state fiscal pressure.
I think this is a pathbreaking use of the information from historical data from Ottoman tax registers in a way that sheds light on human niche construction broadly. The authors have competently extracted the relevant data, analyzed it with state of the art techniques, and interpreted results appropriately.

Remaining questions and concerns that would be good to consider to eliminate confusion:
1. Were there any mixed-ethnic villages?
2. Could the shift be coming from a change in tax rates under Ottoman rule (e.g., towards more uniform rates across the region)?
3. Could the shift be coming from changes in price?
4. How long did it take to establish a vineyard? Something that can be established within a couple of generations as the argument requires?
5. Distance to nearest harbor an drivers: rather than Euclidian distance, wouldn’t an estimate that considers differences in travel cost and altitude variation be more reasonable?

Reviewer #2

(Remarks to the Author)
This manuscript offers a methodologically innovative analysis of changes in agroeconomies and agropolitics at the onset of Early Capitalism in the northern Peloponnesus. Drawing on taxation registers, the authors provide a detailed perspective on socio-economic transformations during this highly dynamic period. The paper is both innovative and engaging, appealing to a broad multidisciplinary readership while addressing key questions in early modern history in the framework of human niche construction theory. I recommend the article for publication, pending revisions.

My main concern relates to what appears in my opinion to be an oversimplification of agro-pastoral economies and, more broadly, sociocultural systems. While some degree of simplification is inherent in the creation of explanatory models, I believe the authors should provide a more nuanced discussion to acknowledge the complexities involved. In discussing the primary diachronic trend, the authors state: “what we see is … a change in how villagers make decisions that shape their ecological niche: instead of relying on social learning (Albanian or Greek ethnic identities determining knowledge inherited from previous generations), they responded to what economic and environmental potential their local environment represented in the newly emergent capitalist conditions” (Lines 236–240). This idea is reiterated in other sections of the

paper. While not challenging the authors' central argument, I suggest that the following points be considered: (i) Social learning should not be assumed to operate solely within ethnic boundaries; (ii) Viticulture involves specialized knowledge, critical for both cultivation and processing; (iii) Cash-crop-oriented production requires integration into preexisting market structures, which are likely to be socially embedded; (iv) Perennial fruit crops represent a form of long-term agricultural investment.

--

Line 49-50. “Prehistoric times or premodern communities”. Perhaps simply “premodern communities? Communities in prehistoric times are by definition premodern.

Line 51. “Much of human niche construction of direct relevance for the current critical condition”. What is it understood here by “direct relevance”? Please clarify in the sentence

Line 52. “Involved advanced agriculture”. What is here understood by advanced agriculture? Please provide explanation and references. Furthermore, the authors are neglecting a discussion of the switch from biomass to coal as main energy source, which is historically key in the making of the “current conditions of the planet”.

Line 55-56. The authors might consider avoiding a reference to the Late Holocene, which boundary is conventionally (IUGS) at 4.2k BP.

Line 58. “However, such studies”. Please provide reference of such studies.

Line 59. The authors might want to explain what it is understood by “socio-ecological” data.

Line 111. “villagers’ cultural values”. Please explain what it is here understood by “cultural values”

Lines 115-117 and 119-121. The authors might want to elaborate further on why the regard viticulture to be proxy for a mixed economy. Grapes are by definition a cash crop, as discussed in later sections of the manuscript, which can reasonably also represent a from of specialized farming.

Lines 153. “distance to the nearest river/stream (crucial for cereal cultivation)”. The authors need to explain why river/stream are crucial for cereal farming. Is arable land irrigated? Are cereals rainfed? More in general, the paper would benefit from a brief discussion of the actual farming systems in the region here considered.

Lines 155-156. “altitude (determining potential for vine cultivation)”. Please, provide explanations of why altitude define viticultural potential.

Lines 163. “suggesting that our independent variables were losing their explanatory power over time”. Is there an explanation for this? Perhaps social integration between ethnic groups? Economic integration?

Lines 202-203. We expected that closed proximity to harbor encouraged cultivation of the vine”. Does this expectation build exclusively on the authors’ reasoning, or on examples from elsewhere? Is topography a factor to consider? Soil drainage? Mountain areas being better suited to grapevine farming on slopes terraces?

219-220. “This wider multi-continental context of the Mediterranean”. What is it understood here by multi-continental context of the Mediterranean? Is this something different that the historical interconnectivity of the Mediterranean basin?

Lines 236-240. Social learning is not ethnically bounded. This requires to say the least a discussion.

Figure 2. In this figure and elsewhere in the manuscript there is an implicit equation of “ethnicity” with “culture”, which is problematic. Please, revise accordingly.

Reviewer #3

(Remarks to the Author)
Summary:
In this paper, the authors apply machine learning techniques to analyze historical land-use decision-making in the Northern Peloponnese at a fine-grained spatio-temporal scale. Their analysis draws on Ottoman taxation records from the 15th and 16th centuries as proxies for land-use practices, correlating these with environmental, ethnic, and cultural variables to understand how decision-making varied over time. A significant contribution of this work is its novel historical dataset, which required extensive effort to develop through geolocation, translation, and the identification of effective variable proxies. The authors have made this valuable resource publicly available for future fine-grained research.

However, we have significant concerns about the theoretical foundations of this research and how they inform the modeling choices. The manuscript requires substantial revisions to address three key issues: the causal framework, the theoretical application of niche construction, and the selection and interpretation of proxy variables. Additionally, while we appreciate the authors’ commitment to open data, the implementation needs improvement to ensure reproducibility.

Major Concerns:

Causal analysis

The manuscript appears to conflate correlative analysis with causal inference. While the authors present a directed acyclic graph (DAG) depicting hypothesized causal relationships, their analytical approach—the BMSC algorithm—is essentially a model selection technique similar to stepwise regression. This approach can identify variables associated with the dependent variable but cannot establish causation. The frequent references to “causality” throughout the paper are therefore misleading and unsupported by the methodology.

Several critical issues need to be addressed. First, high predictive performance or explained variance does not equate to causal relationships, particularly in complex adaptive systems where multiple factors interact. Second, the analysis overlooks crucial statistical considerations: spatial and temporal autocorrelation are not accounted for, potential random effects at the village level are ignored, and the bounded nature of the percentage outcome variable (constrained between 0 and 1) requires special statistical treatment. A more appropriate approach might employ multilevel regression models, generalized linear models, or generalized additive models—all of which could maintain a Bayesian framework using established tools like the brms R package.

While we do not insist that the authors abandon BMSC entirely, they must provide stronger justification for choosing this method over more standard and flexible approaches that are well-suited to this type of analysis. The paper needs to either demonstrate how their approach establishes causality or reframe the analysis as correlative rather than causal. If the authors wish to maintain a causal framework, they need to explicitly address the assumptions and limitations of their approach and provide much more rigorous justification for how their methodology supports causal inference. Alternatively, the paper could be productively reframed as an exploratory analysis of historical land-use patterns, which would better align with the actual strengths of their methodology.

Use of niche construction

The manuscript’s reliance on niche construction theory appears both unnecessary and theoretically misaligned with the actual research conducted. While the documented shifts in land use practices from wheat cultivation to wine production are interesting and well-supported by the data, framing this through niche construction theory adds complexity without analytical value. The authors’ core research questions about changing agricultural strategies and their environmental and socio-economic drivers stand on their own merits.

The paper fails to demonstrate how niche construction theory—which primarily concerns how organisms modify their environment in ways that affect their evolutionary trajectory—meaningfully informs this analysis of agricultural decision-making. Indeed, the authors’ own findings seem to contradict traditional niche construction dynamics: they document communities abandoning established agricultural niches (wheat cultivation) in favor of alternative strategies (wine production) that required different environmental conditions. This suggests that rather than communities becoming more invested in and adapted to their constructed niches over time, they demonstrated considerable flexibility in changing strategies based on economic incentives.

We recommend either removing the niche construction framework entirely or substantially revising the theoretical discussion to clearly articulate how this concept advances our understanding of the observed land use transitions beyond what simpler frameworks of agricultural change and economic decision-making would provide. The strength of this research lies in its novel dataset and careful documentation of agricultural strategy shifts—theoretical frameworks should clarify rather than obscure these contributions.

Selection and interpretation of proxy variables

The selection, justification, and interpretation of proxy variables raise several fundamental concerns that need to be addressed. While the authors provide a causal model diagram, its relationship to the empirical analysis remains inadequately explained and theoretically problematic.

First, the validity of the primary outcome variable—vineyard taxation rate as a proxy for wine-focused land use strategies—requires stronger justification. The authors use the percentage of vineyard taxes relative to total village taxes, but this relationship may not be linear or straightforward. Critical questions remain unanswered: Were wheat and wine taxed at comparable rates? Was taxation based on volume, weight, or value? Are all practitioners of mixed agriculture represented by this vineyard taxation rate? The supplementary materials (Text S1 and S2) suggest these relationships may be more complex than the authors assume.

Second, the characterization of agricultural strategies as opposing rather than complementary requires reconsideration. The causal model implies mutual exclusivity between sheep grazing/wheat growing areas and grape-growing regions, but this dichotomy may be artificial. The analysis should consider whether these strategies might instead represent different points on an intensification-extensification spectrum, particularly given differences in storage requirements, labor inputs, and seasonal timing.

Third, crucial contextual information appears to be missing from the manuscript. For instance, the authors do not explicitly discuss how Greek communities had millennia of wine-making tradition while Albanian migrants brought different agricultural preferences oriented toward wheat and pastoral farming. This historical context, which we discovered only through external sources (a recorded presentation by Dr. Izdebski), seems essential for understanding the patterns in the

data. The terminology used to describe these strategies—“mixed” versus “wheat- and pastoral-focused agriculture”—adds further confusion. The latter term itself implies a mixed strategy, making the distinction unclear.

Open-data and script availability
We appreciate the authors’ commitment to open science principles, demonstrated by making their novel dataset available as an Excel spreadsheet with a DOI and providing access to the BMSC algorithm through a web application. These are valuable steps toward research transparency. We have a few suggestions to further enhance the reproducibility and reusability of this work.

The current materials could be strengthened by providing clearer documentation connecting the dataset to the analytical workflow. While the BMSC web application is a useful tool, brief instructions for using it with the provided dataset would help other researchers build upon this work. We recognize that full computational reproducibility requires significant additional effort, but even basic documentation of the key analytical steps would be valuable for the research community.

If feasible, we recommend considering these additions in future versions:
- Basic documentation of any pre-processing steps applied to the raw data
- Simple instructions for reproducing key results
- Notes about the computational environment used for the analysis

We look forward to seeing this valuable research continue to develop. The unique historical dataset alone is an important contribution to the field, and greater accessibility of the analysis tools will only enhance its impact.

Reviewer #4

(Remarks to the Author)
I co-reviewed this manuscript with one of the reviewers who provided the listed reports. This is part of the Nature Communications initiative to facilitate training in peer review and to provide appropriate recognition for Early Career Researchers who co-review manuscripts.

Version 1:

Reviewer comments:

Reviewer #1

(Remarks to the Author)
I believe the authors have done a good job of addressing the questions and concerns that I expressed about the original submission.

Reviewer #2

(Remarks to the Author)
The points previously raised have been addressed by the authors. Pending some minor revisions, I would recommend acceptance of the paper for publication.

• Lines 51–54: The statement “What sets human activities apart from those of all other species is their deeply sociocultural nature, which becomes even more evident and complex with the progress of human cultural evolution and the emergence of modern complex societies” is too generic. Please revise to make the point more specific and substantiated.

• Line 57–58: “Such enrichment of our perspective has the double advantage of studying societies that are more similar to our own”. Please expand on how “similarity” is understood in this context.

• Line 91: “dye-producing plants”. Please specify the main dye plants referred to here.

• Lines 145–151: Crop-tree agriculture is equated with viticulture. Does olive cultivation play a role? Which other crop trees are considered relevant? Please specify.

Reviewer #3

(Remarks to the Author)
We appreciate the authors' detailed responses and clarifications, particularly regarding the niche construction theoretical framework. The authors have adequately addressed most of our methodological and theoretical concerns from the initial review. Their reframing of the analysis as more exploratory and the clearer distinction between correlation and causation strengthen the manuscript considerably.

However, the authors did not address our concerns about computational reproducibility and data access. The GitHub repository appears to link to the general BMSC method source code rather than the specific application to this dataset. The

Pandora repository link does not appear publicly accessible and needs verification. Even if full computational reproducibility isn't feasible, basic documentation of the analytical workflow, pre-processing steps, and instructions for using the BMSC tool with the provided dataset would significantly enhance accessibility for other researchers.

Reviewer #4

(Remarks to the Author)
I co-reviewed this manuscript with one of the reviewers who provided the listed reports. This is part of the Nature Communications initiative to facilitate training in peer review and to provide appropriate recognition for Early Career Researchers who co-review manuscripts.

Version 2:

Reviewer comments:

Reviewer #2

(Remarks to the Author)
All the questions and concerns previously raised have been satisfactorily addressed by the authors

(Remarks on code availability)

Reviewer #3

(Remarks to the Author)
The code and data release is now accessible and looks great. I am satisfied with the current state of the manuscript and its supporting materials.

(Remarks on code availability)

Reviewer #4

(Remarks to the Author)
I co-reviewed this manuscript with one of the reviewers who provided the listed reports. This is part of the Nature Communications initiative to facilitate training in peer review and to provide appropriate recognition for Early Career Researchers who co-review manuscripts.

(Remarks on code availability)

Open Access This Peer Review File is licensed under a Creative Commons Attribution 4.0 International License, which permits use, sharing, adaptation, distribution and reproduction in any medium or format, as long as you give appropriate credit to the original author(s) and the source, provide a link to the Creative Commons license, and indicate if changes were made.
In cases where reviewers are anonymous, credit should be given to 'Anonymous Referee' and the source.
The images or other third party material in this Peer Review File are included in the article's Creative Commons license, unless indicated otherwise in a credit line to the material. If material is not included in the article's Creative Commons license and your intended use is not permitted by statutory regulation or exceeds the permitted use, you will need to obtain permission directly from the copyright holder.
To view a copy of this license, visit https://creativecommons.org/licenses/by/4.0/

Reply to reviews

Reviewer #1

This paper uses data from three tax registers of the Ottoman Empire from the15th-16th centuries to study human niche construction in a historical context, specifically to compare the evolution of land use choices of Albanian and Greek village communities in southern Greece over time. To estimate the effect of ethnicity, the authors apply a Bayesian machine learning algorithm to a geospatial quantitative dataset. They find that under Ottoman rule these communities experienced a significant economic transition. Whereas Greek/Albanian ethno-cultural identities previously played a major role in determining local niche construction strategies, successive generations under Ottoman rule shifted to strategies based on the environmental characteristics of their surroundings and the investment potential of each village community. The authors interpret this phenomenon as being a product of the transition from unstable political conditions and limited commodification of agriculture to lasting peace, intense market integration and strong state fiscal pressure.
I think this is a pathbreaking use of the information from historical data from Ottoman tax registers in a way that sheds light on human niche construction broadly. The authors have competently extracted the relevant data, analyzed it with state of the art techniques, and interpreted results appropriately.

Thank you for your encouraging comments!

Remaining questions and concerns that would be good to consider to eliminate confusion:
1. Were there any mixed-ethnic villages?
The only mixed ethnic village was Martina der Nokastro, as recorded in TT10 (1460s) comprising 10 Greek and 5 Albanian households. By TT80 (1510s), the village had expanded to 33 households, predominantly Greek, with the exception of possibly one Albanian household (Gin < Gjin [=John]). In TT607 (1580s), the village comprised 29 households, all Greek with a possible exception of two households headed by two certain 'Miho' (Albanian diminutive of Mëhill [=Michael]). For our analysis, we have chosen to consider only the Greek population data from TT10, as it appears that the Greek community became the predominant ethnic group in the village during the 16th century. We included this information in the methods section.

2. Could the shift be coming from a change in tax rates under Ottoman rule (e.g., towards more uniform rates across the region)?

In general, the Ottoman administration was interested in taxing the greatest possible variety of economic activities in ways that were flexible and adapted to local conditions. For instance, the use of local measurement units in TT80 (1510s) suggests a pragmatic/realistic approach to taxation, indicating clear regional differences within the Province of the Morea (entire Peloponnese). In contrast, TT10 (1460s), compiled during wartime, likely used a more general/uniform and rushed recording method, since the surveyors were assigned to assess the revenues in villages and fortified towns that frequently shifted between Ottoman and Venetian control over just a couple of months. Thus, in general, the taxation system tended to become more diverse with time, but in general it remained stable over the entire study period, based on cash payments of the tax, which indeed ushered peasants into commodification and market economy. The only meaningful change relevant to our study was the removal of the reduction of the capital (poll) tax for the Albanians (*ispence*), which was 20% in the first cadaster (1460s). Thus, for the Albanians it was indeed a tax increase and the incentive to acquire more cash to meet the fiscal demands. In fact, contrary to the Byzantines or Venetians, the Ottoman authorities were very apt at pacifying semi-nomadic communities, such as

Turkomans or Albanians, and made significant efforts at integrating them into the broader socio-political system. We now pointed this out in the discussion.

3. Could the shift be coming from changes in price?
Yes, we believe it is partly coming from that – there is increasing price pressure (rising absolute price of wheat and wine in grams of silver) as displayed on Fig. 4. This is what we were referring to when discussing market pressures and incentives, which we now made clearer in the discussion.

4. How long did it take to establish a vineyard? Something that can be established within a couple of generations as the argument requires?
3–5 years (modern times). This could also hold true for the pre-industrial period, given that TT10 (1460s) records vineyards held in the personal demesne of the newly established timariots (1461–63), which yielded a revenue of 80,359 *akçe*, appr. 1/3 of the total viticulture tax (tax on vineyards cultivated by the villagers: 163,127 *akçe*). (Liakopoulos 2009, 207 – reference 24). It was a major investment (land formation into terraces or plantations, planting of vines, building supports), but it provided a relatively quick return on invested capital compared, for instance, to fruit trees, in particular olive groves (where the return would require at least 20 years).

5. Distance to nearest harbor an drivers: rather than Euclidian distance, wouldn't an estimate that considers differences in travel cost and altitude variation be more reasonable?
We considered these issues when carrying out our study, prior to submission. Based on the available primary data from the Ottoman Empire, there was no way of estimating the transportation cost ; the data are too fragmentary and varied across the regions of the Empire (e.g., we cannot take a single piece of information about transportation cost in Anatolian mountains to represent the Peloponnese). However, we believe the assumption of uniform transportation costs across our relatively constrained study region is reasonable. Therefore, we looked into distance and altitude variation as physical factors determining the transport cost, and we calculated both values for each village, which in particular for the altitude difference was a time-consuming process. However, it turned out that the altitude variation is highly correlated with the altitude itself, and hence we opted not to include it in the paper at all. In response to the reviewer question, we now provide this data in the supplementary dataset and in the methods as well as the independent variable correlation table.

Reviewer #2

This manuscript offers a methodologically innovative analysis of changes in agroeconomies and agropolitics at the onset of Early Capitalism in the northern Peloponnesus. Drawing on taxation registers, the authors provide a detailed perspective on socio-economic transformations during this highly dynamic period. The paper is both innovative and engaging, appealing to a broad multidisciplinary readership while addressing key questions in early modern history in the framework of human niche construction theory. I recommend the article for publication, pending revisions.

Thank you for your encouraging comments!

My main concern relates to what appears in my opinion to be an oversimplification of agro-pastoral economies and, more broadly, sociocultural systems. While some degree of simplification is inherent in the creation of explanatory models, I believe the authors should provide a more nuanced discussion to acknowledge the complexities involved. In discussing the primary diachronic trend, the authors state: "what we see is … a change in how villagers make decisions that shape their ecological niche: instead of relying on social learning (Albanian or Greek ethnic identities determining knowledge inherited from previous generations), they responded to what economic and

environmental potential their local environment represented in the newly emergent capitalist conditions" (Lines 236–240). This idea is reiterated in other sections of the paper.

We now modified this sentence to be more precise and avoid unnecessary assumptions (simplifications). We also reviewed the entire paper to offer a more nuanced discussion.

While not challenging the authors' central argument, I suggest that the following points be considered:

(i) Social learning should not be assumed to operate solely within ethnic boundaries

We agree, and now refer to cross-boundary social learning too. However, there is evidence that most of agriculture/ecology-related social learning within the peasant households of the 14th-15th c. Southern Greece actually operated within ethnic boundaries. We now avoid unnecessary generalization in the text.

(ii) Viticulture involves specialized knowledge, critical for both cultivation and processing

We agree, and we assume there must have been a point when the Albanian peasants acquired this knowledge somewhere between the 1460s and the 1510s. We have no direct record of how the Albanians accessed the required know-how, but we hypothesize that the timariots – noble landholders – were among the key agents of this change. We now include this point in the discussion within the main text.

(iii) Cash-crop-oriented production requires integration into preexisting market structures, which are likely to be socially embedded

Indeed, and the Greeks already had such access to market structures, also because there was a phase of Venetian commodified agriculture in this part of the Peloponnese beforehand (13th-14th c. CE). The Albanians were isolated from these market structures as new settlers, living in more distant settlements with less access to Greek (!) market towns. It was the Ottoman cash-based taxation system – that also equaled Greeks and Albanians over time – that at least partly forced them to connect to these market structures.
The majority of the Albanian settlements, 233 (57.24%), consisted of 1–20 households, whereas the Greeks tended to reside in larger towns. The regional capitals were inhabited by Greeks only; the Jewish presence in Corinth constitutes the only exception to this pattern. The average number of families residing in Greek villages was 41.29 and for the Albanian counterpart the average was 11.86; hence, the average Greek village was approximately three and a half times larger than the Albanian one (Liakopoulos, 2019).

(iv) Perennial fruit crops represent a form of long-term agricultural investment

Yes, we agree. We now emphasize this point in the revised text of the manuscript.

Line 49-50. "Prehistoric times or premodern communities". Perhaps simply "premodern communities? Communities in prehistoric times are by definition premodern.

Changed to "prehistoric or isolated", as part of a broader rewriting of the introduction.

Line 51. "Much of human niche construction of direct relevance for the current critical condition". What is it understood here by "direct relevance"? Please clarify in the sentence

Text removed, as part of a broader rewriting of the introduction, to address this and other concerns of Reviewers # 2 and #3.

Line 52. "Involved advanced agriculture". What is here understood by advanced agriculture? Please provide explanation and references. Furthermore, the authors are neglecting a discussion of the switch from biomass to coal as main energy source, which is historically key in the making of the "current conditions of the planet".

Text removed, as part of a broader rewriting of the introduction, to address this and other concerns of Reviewers # 2 and #3.

Line 55-56. The authors might consider avoiding a reference to the Late Holocene, which boundary is conventionally (IUGS) at 4.2k BP.

Text removed, as part of a broader rewriting of the introduction, to address this and other concerns of Reviewers # 2 and #3.

Line 58. "However, such studies". Please provide reference of such studies.

Text removed, as part of a broader rewriting of the introduction, to address this and other concerns of Reviewers # 2 and #3.

Line 59. The authors might want to explain what it is understood by "socio-ecological" data.

Text removed, as part of a broader rewriting of the introduction, to address this and other concerns of Reviewers # 2 and #3.

Line 111. "villagers' cultural values". Please explain what it is here understood by "cultural values"

Text removed, as part of a broader rewriting of the introduction, to address this and other concerns of Reviewers # 2 and #3.

We thank the reviewer for this and earlier comments on the introductory part of our paper, as this helped us to streamline and clarify our argument, also avoiding unnecessary confusion or referring terms that are not critical for making our points and demonstrating the broader implications/relevance of our work.

Lines 115-117 and 119-121. The authors might want to elaborate further on why the regard viticulture to be proxy for a mixed economy. Grapes are by definition a cash crop, as discussed in later sections of the manuscript, which can reasonably also represent a from of specialized farming.

Inspired by the comments of this Reviewer and Reviewer # 3, we decided to drop the juxtaposition of the Greek "mixed" framing and Albanian "simple" farming. In fact, all farming activities could result in selling products at markets and obtaining cash – grain, in particular wheat, was a cash crop too, as were the animal products (as textiles or meat). Thus, the question was not of switching between types of cultivation that could be commodified or not, but rather by giving up earlier traditional ethnicity-related ways of doing things and adapting the "cultivation portfolio" of a household and a village to maximize cash inflow, necessary to meet the fiscal demands and survive successfully in the context of a deeply monetized, commodified rural economy.

Lines 153. "distance to the nearest river/stream (crucial for cereal cultivation)". The authors need to explain why river/stream are crucial for cereal farming. Is arable land irrigated? Are cereals rainfed? More in general, the paper would benefit from a brief discussion of the actual farming systems in the region here considered.

As described in Table 1 (which follows at the end of the paper in its current submission format), "Riverbeds and areas around streams are characterized by more moisture in the autumn and their

flattened morphology encouraged grain cultivation and discouraged vine cultivation.”. Irrigation was not the main reason for riverbeds being more attractive for cereal cultivation – in the 1810s-20s, only 19.5% of the cultivated lands in the entire Peloponnese in the 1810s–20s were irrigated (Asdrachas 2003, 212).

We added the requested paragraph to the introduction.

Lines 155-156. “altitude (determining potential for vine cultivation)”. Please, provide explanations of why altitude define viticultural potential.

This is explained in Table 2. This was probably related to the type of soils and availability of sunshine, both strongly correlated with the altitude in the case of our study region, thus altitude represents the generic environmental potential of each village’s environs. This was also recognised by the contemporaries. When mapping the vineyards held in timariots’ personal demesne (*ḫāṣṣa*) and factoring in their altitude, it is observed that mountainous *ḫāṣṣa* vineyards yield an average production value of 558.8 *aķçe*s per *dönüm* (919.3 m^2), whereas lowland and semi-mountainous vineyards produce 480.68 and 479.16 *aķçe*s per *dönüm*, respectively (Liakopoulos, 2009). This disparity in values must not be attributed to differing vineyard efficiencies, as viticultural principles dictate the opposite: lowland vineyards are considerably more productive than their mountainous counterparts. It is plausible that the mountainous vineyards yielded a higher quality of must – or of higher market value –, which subsequently incurred a greater tax rate. Basically, wine from the mountains was better and could bring more cash. We now expanded the information given in Table 2.

Lines 202-203. We expected that closed proximity to harbor encouraged cultivation of the vine”. Does this expectation build exclusively on the authors’ reasoning, or on examples from elsewhere? Is topography a factor to consider? Soil drainage? Mountain areas being better suited to grapevine farming on slopes terraces?

As described in Table 2, we expected that better access to transport opportunities – easier access to markets – would incentivize even further income-oriented optimization of ecological choices characteristic for (early) capitalist societies. We now describe this point point in the main text, with a reference to relevant research.

219-220. “This wider multi-continental context of the Mediterranean”. What is it understood here by multi-continental context of the Mediterranean? Is this something different that the historical interconnectivity of the Mediterranean basin?

We mean period-specific degree of interconnectivity of the Mediterranean basin, that is its integration into a single market, as we already explained in the second clause of the same sentence and we now tried to make even clearer.

Lines 236-240. Social learning is not ethnically bounded. This requires to say the least a discussion.

We agree with the general point (social learning is not *per se* ethnically bounded), but we still believe there is convincing evidence – also that presented in the supplementary text – that in our case study, in the earlier phase of the 14th-15th c. CE, following the Albanian migration, most if not all of agriculture- and livelihood-related social learning actually took part within the boundaries of the ethnic communities. We adjusted the sentence to make it more precise and avoid confusion.

Figure 2. In this figure and elsewhere in the manuscript there is an implicit equation of “ethnicity” with “culture”, which is problematic. Please, revise accordingly.

We have revised this aspect across the manuscript, although during the 14th-15th c. CE in the southern Greek lands cultural identities (ways of life, material culture, diet, religious traditions, etc.) were actually very strongly associated with ethnicity. As we also described in the manuscript, this association persisted for several more centuries in many domains, yet not in the ecological niche construction choices. We did not change Figure 2, because with the revised text of the paper we believe the figure no longer implies equation of culture with ethnicity, and it is clear ethnicity is a cultural variable – part of broader phenomenon that culture is – that happens to be represented in our data.

Reviewer #3

Summary:
In this paper, the authors apply machine learning techniques to analyze historical land-use decision-making in the Northern Peloponnese at a fine-grained spatio-temporal scale. Their analysis draws on Ottoman taxation records from the 15th and 16th centuries as proxies for land-use practices, correlating these with environmental, ethnic, and cultural variables to understand how decision-making varied over time. A significant contribution of this work is its novel historical dataset, which required extensive effort to develop through geolocation, translation, and the identification of effective variable proxies. The authors have made this valuable resource publicly available for future fine-grained research.

However, we have significant concerns about the theoretical foundations of this research and how they inform the modeling choices. The manuscript requires substantial revisions to address three key issues: the causal framework, the theoretical application of niche construction, and the selection and interpretation of proxy variables. Additionally, while we appreciate the authors' commitment to open data, the implementation needs improvement to ensure reproducibility.

Major Concerns:

Causal analysis
The manuscript appears to conflate correlative analysis with causal inference. While the authors present a directed acyclic graph (DAG) depicting hypothesized causal relationships, their analytical approach—the BMSC algorithm—is essentially a model selection technique similar to stepwise regression. This approach can identify variables associated with the dependent variable but cannot establish causation. The frequent references to "causality" throughout the paper are therefore misleading and unsupported by the methodology.

We agree with the reviewer that BMSC is not a causality tool and as such a clearer separation between causation and correlation is now reflected in the manuscript's text.

Several critical issues need to be addressed. First, high predictive performance or explained variance does not equate to causal relationships, particularly in complex adaptive systems where multiple factors interact. Second, the analysis overlooks crucial statistical considerations: spatial and temporal autocorrelation are not accounted for, potential random effects at the village level are ignored, and the bounded nature of the percentage outcome variable (constrained between 0 and 1) requires special statistical treatment. A more appropriate approach might employ multilevel regression models, generalized linear models, or generalized additive models—all of which could maintain a Bayesian framework using established tools like the brms R package.

On the first point, BMSC identifies candidate predictors correlated with the outcome, which might indeed include confounders or mediators. We addressed this by more clearly separating in the text the exploratory study of variable association from how this may inform our proposed causal hypothesis.

On the second point, the analysis was performed on three separate time bins and there is only one data point per village per time bin. For these reasons, temporal autocorrelation and random effects at the village level are ignored. We tested for spatial autocorrelation but this was not significant in two time bins (1510s and 1580s) and weak for the 1460s time bin. Finally, on the constrained outcome variable, for each time bin we compared observed and predicted outcomes. There is no prediction outside 0 to 100% for two time bins (1510s and 1580s) while for the 1460s time bin only 6% of the predictions are slightly below 0%.

We have modified the methods section to summarize the above and included in the SI spatial autocorrelation analysis and observed vs. prediction comparisons.

While we do not insist that the authors abandon BMSC entirely, they must provide stronger justification for choosing this method over more standard and flexible approaches that are well-suited to this type of analysis. The paper needs to either demonstrate how their approach establishes causality or reframe the analysis as correlative rather than causal. If the authors wish to maintain a causal framework, they need to explicitly address the assumptions and limitations of their approach and provide much more rigorous justification for how their methodology supports causal inference. Alternatively, the paper could be productively reframed as an exploratory analysis of historical land-use patterns, which would better align with the actual strengths of their methodology.

We have now reframed the analysis as more exploratory. Please note that there is no assumption-free method for causal inference; every approach trades identifiability for theoretical constraints. The same would apply to our own proposed DAG and we must consider the difficulties of modeling complex adaptive systems compounded by the use of observational historical data. For instance, we cannot exclude that there are missing confounders (see R2 values for BMSC analysis) and that actual causal structures may differ considerably from our DAG. BMSC can identify useful variable associations which could be relevant to propose different hypotheses. We now comment how the result align with our proposed hypothesis which is supported by broader considerations of emergent capitalism.

Use of niche construction

The manuscript's reliance on niche construction theory appears both unnecessary and theoretically misaligned with the actual research conducted. While the documented shifts in land use practices from wheat cultivation to wine production are interesting and well-supported by the data, framing this through niche construction theory adds complexity without analytical value. The authors' core research questions about changing agricultural strategies and their environmental and socio-economic drivers stand on their own merits.

Thank you for this positive evaluation of our research. However, we believe that the niche construction theory both helps to frame better the changes we observe and also that our research does contribute to niche construction theory (as observed by Reviewer # 1), in particular to the research on sociocultural niche construction.

The paper fails to demonstrate how niche construction theory—which primarily concerns how organisms modify their environment in ways that affect their evolutionary trajectory—meaningfully informs this analysis of agricultural decision-making.

Please note that the evolutionary trajectory concerns both selection at the genetic level, as well as the changes in the cultural and ecological inheritance. It is this interplay of the cultural and ecological inheritance, and in particular the complexity and autonomy of the human sociocultural niche, that our paper addresses and documents with new evidence. We now make it more clear and nuanced in the new introduction that was inspired by this Reviewer's comments, for which we are indeed grateful.

Indeed, the authors' own findings seem to contradict traditional niche construction dynamics: they document communities abandoning established agricultural niches (wheat cultivation) in favor of alternative strategies (wine production) that required different environmental conditions. This suggests that rather than communities becoming more invested in and adapted to their constructed niches over time, they demonstrated considerable flexibility in changing strategies based on economic incentives.

Exactly this fact documented by our research on sociocultural niche construction in complex human societies in this paper is the key finding of our study that has bearing on the debates on (human) niche construction theory. Human communities (Albanian and Greek peasants) were responsive to rapidly changing and cascading pressures within the sociocultural niche by modifying their ecological niche – we now emphasize and discuss it even more in the introduction and discussion.

We recommend either removing the niche construction framework entirely or substantially revising the theoretical discussion to clearly articulate how this concept advances our understanding of the observed land use transitions beyond what simpler frameworks of agricultural change and economic decision-making would provide. The strength of this research lies in its novel dataset and careful documentation of agricultural strategy shifts—theoretical frameworks should clarify rather than obscure these contributions.

Following this recommendation we substantially revised the niche construction framework of our paper and wrote a new introduction to clearly articulate how this concept advances the understanding of our results and how our results contribute to the nuancing and further development of this concept. Thank you for your feedback and encouragement!

Selection and interpretation of proxy variables

The selection, justification, and interpretation of proxy variables raise several fundamental concerns that need to be addressed. While the authors provide a causal model diagram, its relationship to the empirical analysis remains inadequately explained and theoretically problematic.

We have now addressed these issues – please see below, as well as our answers to Reviewer # 2.

First, the validity of the primary outcome variable—vineyard taxation rate as a proxy for wine-focused land use strategies—requires stronger justification. The authors use the percentage of vineyard taxes relative to total village taxes, but this relationship may not be linear or straightforward. Critical questions remain unanswered:

Were wheat and wine taxed at comparable rates?

Wheat and wine were taxed at comparable rates, with taxes related to vineyard involving a more flexible and less predictable – potentially risky for taxpayers – system of estimating and collecting taxes. From the available evidence it is clear that the Ottoman state did everything it could to tax all aspects of vine cultivation and wine production, and thus to tap into this lucrative business and derive a substantial fiscal incomes from it. Consequently, in no way vine cultivation a tax-evasive activity compared to grain cultivation or animal herding.

More specifically, In the Ottoman fiscal system, apart from the poll tax paid in cash, the approach to agricultural tithe collection was contingent upon the specific crop. While the tithe on cereals and legumes was consistently rendered in kind – representing a direct portion of the harvest – tax collectors exercised discretion in determining the payment method for vineyard taxes, allowing for either in-kind or monetary remittance and often following pre-Ottoman practice. Viticulture, as a taxable activity, encompassed a range of levies that also extended to the process of vinification. Within the registers examined, these included taxes on vineyards and vineyard land (whether held by villagers or within the personal demesne of timariots), must, span (tax on must put in barrel for fermentation), wine, and raisins. The granular detail, localised differentiation, and comprehensive nature of this taxation system suggest a deliberate strategy designed to mitigate potential tax evasion within the viticultural sector. The preeminent role of viticulture in the calculation of monetary fief (*tīmār*) income, and consequently, in the imputed value of agricultural output, was directly correlated with wine prices.

Was taxation based on volume, weight, or value?

While for grain the taxation was based on weight (a 10% in kind tax), for vine cultivation it was much more diversified, depending on the period and region of the empire, with the clear goal of raising as much tax as possible. The Ottoman administration employed a range of measurement units for assessing agricultural harvests and their corresponding tax liabilities, frequently aligning with established local practices for pragmatic reasons. In the case of cereals, assessment was based mostly on weight, with distinct standards for wheat compared to other grains. Additionally, the *ḥiml*, a unit of volume typically ranging from 6 to 8 *kile*, was also utilised for measurement of cereal production.

Are all practitioners of mixed agriculture represented by this vineyard taxation rate?

Please note we no longer refer to mixed agriculture as represented by vine tax, but rather discuss the continuum between grain/livestock vs tree crop dominant strategies. In the context of the NW Peloponnese, and contrary to the southern Peloponnese, the other major tree crop, olive, was of minimal significance, and hence it is spectrum between sole focus on grain and herding versus specialization in vine - as represented by the share of vine-related taxes in all cultivation taxes – that best represents the overall diversity of ecological niche construction strategies in our study area.

Moreover, the comprehensive array and meticulous nature of viticulture imposts implemented during the Ottoman period are posited to have significantly constrained opportunities for tax evasion. Nevertheless, historical records indicate recurrent instances of wine smuggling, as attested by sultanic and judicial orders from the *mühimme defterleri* (decisions and decrees issued by the Imperial Council). In the case of our study region, the NW Peloponnese

The supplementary materials (Text S1 and S2) suggest these relationships may be more complex than the authors assume.

We now added Text S3 that clarifies the vine vs grain taxation issues, using the answers provided above.

Second, the characterization of agricultural strategies as opposing rather than complementary requires reconsideration. The causal model implies mutual exclusivity between sheep grazing/wheat growing areas and grape-growing regions, but this dichotomy may be artificial. The analysis should consider whether these strategies might instead represent different points on an intensification-extensification spectrum, particularly given differences in storage requirements, labor inputs, and seasonal timing.

Indeed, we follow now the suggestion of Reviewers # 2 and # 3: instead of juxtaposing simple and mixed agriculture, we discuss the degree of involvement in crop tree cultivation as the best proxy representing the ecological niche construction strategy of a village within a broad spectrum of options (consisting of mixing tree crops with grain/livestock in varying proportions).

Third, crucial contextual information appears to be missing from the manuscript. For instance, the authors do not explicitly discuss how Greek communities had millennia of wine-making tradition while Albanian migrants brought different agricultural preferences oriented toward wheat and pastoral farming. This historical context, which we discovered only through external sources (a recorded presentation by Dr. Izdebski), seems essential for understanding the patterns in the data.

We now extended Supplementary Text S1 to include even more information on this issue and we refer to it explicitly in the introduction. We do not think that any of the presentations that we have given on the research presented in this paper contained relevant contextual information that is absent from the main text or the supplementary. The facts referred to in this Reviewer comment were present in the submission version of the Supplementary Text S1, but indeed they were not mentioned explicitly enough in the main text, for reasons of space constraints.

The terminology used to describe these strategies—“mixed” versus “wheat- and pastoral-focused agriculture”—adds further confusion. The latter term itself implies a mixed strategy, making the distinction unclear.

We removed this terminology and use a different one instead now.

Reviewer #1 (Remarks to the Author):

I believe the authors have done a good job of addressing the questions and concerns that I expressed about the original submission.

Thank you!

Reviewer #2 (Remarks to the Author):

The points previously raised have been addressed by the authors. Pending some minor revisions, I would recommend acceptance of the paper for publication.

• Lines 51–54: The statement "What sets human activities apart from those of all other species is their deeply sociocultural nature, which becomes even more evident and complex with the progress of human cultural evolution and the emergence of modern complex societies" is too generic. Please revise to make the point more specific and substantiated.

Thank you, we now added a sentence that makes it more concrete.

• Line 57–58: "Such enrichment of our perspective has the double advantage of studying societies that are more similar to our own". Please expand on how "similarity" is understood in this context.

Thank you – we have now made it more precise.

• Line 91: "dye-producing plants". Please specify the main dye plants referred to here.

Information provided in the text. Both madder (*Rubia tinctorum L.*) and Aleppo oak (*Quercus infectoria*) are attested in an Ottoman report of the Peloponnese in the early 19th c., see Georgios C. Liakopoulos, «Το κύκνειο άσμα των Οθωμανών στην Πελοπόννησο. Ανώνυμη οθωμανική έκθεση για τον επαναστατημένο Μοριά (πρόδρομη ανακοίνωση)», *Πελοποννησιακά* 32 (2022), 232–33 (229–48). Our 16th c. registers record the scale insect kermes or crimson worm (*Kermes vermilio*), which lives on oaks; a deep red dye is produced from their dried bodies. We did not include this reference in the article for space reasons, however, since it is not relevant for the core argument of the paper.

• Lines 145–151: Crop-tree agriculture is equated with viticulture. Does olive cultivation play a role? Which other crop trees are considered relevant? Please specify.

Thank you. Indeed, the word "olive" is mentioned only once in our entire paper, including the supplementary materials, and this is for a reason. As we explain in the introduction and again with reference to even more secondary literature at the beginning of the results section, vine was indeed the dominant tree-crop in the context of NW Peloponnese, contrary to, for instance, SW Peloponnese with its famous *Kalamata* olives. We also list other crops in the introduction (paragraph three), but these did not produce taxes in such amounts that would be comparable to vine and warrant a separate analysis.

To make it clear we mean the specific conditions of NW Peloponnese, we made changes to the sentence beginning "As vine was the key tree crop in the Mediterranean bioclimatic conditions of the northwestern Peloponnese…" to make this clear.

Reviewer #3 (Remarks to the Author):

We appreciate the authors' detailed responses and clarifications, particularly regarding the niche construction theoretical framework. The authors have adequately addressed most of our methodological and theoretical concerns from the initial review. Their reframing of the analysis as more exploratory and the clearer distinction between correlation and causation strengthen the manuscript considerably.

However, the authors did not address our concerns about computational reproducibility and data access. The GitHub repository appears to link to the general BMSC method source code rather than the specific application to this dataset. The Pandora repository link does not appear publicly accessible and needs verification. Even if full computational reproducibility isn't feasible, basic documentation of the analytical workflow, pre-processing steps, and instructions for using the BMSC tool with the provided dataset would significantly enhance accessibility for other researchers.

Indeed the reviewer is correct and our apologies for the situation. Pandora underwent an upgrade that involved a machine migration and the files were wrongly uploaded to the old machine. We have re-upload these (readme file with instructions on how to run the BMSC interface, model files, and modelling results). This situation was a chance event and since we now paid special attention to avoid it, the link has become permanent and the full reproducibility files are available.

Reviewer #4 (Remarks to the Author):

I co-reviewed this manuscript with one of the reviewers who provided the listed reports. This is part of the Nature Communications initiative to facilitate training in peer review and to provide appropriate recognition for Early Career Researchers who co-review manuscripts.

Thank you!
